# Temporal Dynamic Analysis of Alternative Splicing During Embryonic Development in Zebrafish

**DOI:** 10.3389/fcell.2022.879795

**Published:** 2022-07-08

**Authors:** Zhe Liu, Wei Wang, Xinru Li, Xiujuan Zhao, Hongyu Zhao, Wuritu Yang, Yongchun Zuo, Lu Cai, Yongqiang Xing

**Affiliations:** ^1^ The Inner Mongolia Key Laboratory of Functional Genome Bioinformatics, School of Life Science and Technology, Inner Mongolia University of Science and Technology, Baotou, China; ^2^ State Key Laboratory of Reproductive Regulation and Breeding of Grassland Livestock, College of Life Sciences, Inner Mongolia University, Hohhot, China; ^3^ Digital College, Inner Mongolia Intelligent Union Big Data Academy, Inner Mongolia Wesure Date Technology Co., Ltd., Hohhot, China; ^4^ Hohhot Science and Technology Bureau, Hohhot, China

**Keywords:** zebrafish, embryonic development, gene expression, alternative splicing, splicing factors

## Abstract

Alternative splicing is pervasive in mammalian genomes and involved in embryo development, whereas research on crosstalk of alternative splicing and embryo development was largely restricted to mouse and human and the alternative splicing regulation during embryogenesis in zebrafish remained unclear. We constructed the alternative splicing atlas at 18 time-course stages covering maternal-to-zygotic transition, gastrulation, somitogenesis, pharyngula stages, and post-fertilization in zebrafish. The differential alternative splicing events between different developmental stages were detected. The results indicated that abundance alternative splicing and differential alternative splicing events are dynamically changed and remarkably abundant during the maternal-to-zygotic transition process. Based on gene expression profiles, we found splicing factors are expressed with specificity of developmental stage and largely expressed during the maternal-to-zygotic transition process. The better performance of cluster analysis was achieved based on the inclusion level of alternative splicing. The biological function analysis uncovered the important roles of alternative splicing during embryogenesis. The identification of isoform switches of alternative splicing provided a new insight into mining the regulated mechanism of transcript isoforms, which always is hidden by gene expression. In conclusion, we inferred that alternative splicing activation is synchronized with zygotic genome activation and discovered that alternative splicing is coupled with transcription during embryo development in zebrafish. We also unveiled that the temporal expression dynamics of splicing factors during embryo development, especially co-orthologous splicing factors. Furthermore, we proposed that the inclusion level of alternative splicing events can be employed for cluster analysis as a novel parameter. This work will provide a deeper insight into the regulation of alternative splicing during embryogenesis in zebrafish.

## 1 Introduction

Zebrafish (*Danio rerio*) is a prominent vertebrate model organism and has been extensively used to study embryonic development because of its high fecundity, external embryogenesis, rapid embryonic development, and nearly transparent embryo (Nudelman et al., 2018). In the past decades, many studies focused on embryogenesis of zebrafish have been published. Mathavan et al. (2005) characterized the first gene expression profile using microarrays from an unfertilized egg to 2 days post-fertilization. White et al. (2017) profiled mRNA expression of zebrafish development using RNA-seq across 18 time points from one cell to 5 days post-fertilization. The non-coding RNAs were identified systematically during zebrafish embryogenesis (Pauli et al., 2012; Wei et al., 2012; Yao et al., 2014). The dynamic analyses of transcription and epigenetics during zygotic genome activation (ZGA) were conducted *via* multi-omics technologies (Vastenhouw et al., 2010; Aanes et al., 2013; Harvey et al., 2013; Potok et al., 2013; Heyn et al., 2014; Zhang et al., 2014; Lee et al., 2013; Zhao et al., 2017). The gene expression mapping and developmental trajectories during zebrafish embryogenesis were reconstructed by scRNA-seq (Farrell et al., 2018; Wagner et al., 2018). Although previous studies on the early embryonic development of zebrafish have provided important biological insights, the regulatory mechanisms of embryogenesis in zebrafish have not been completely revealed, especially the effect of alternative splicing (AS) in this process.

Alternative splicing is a ubiquitous and conserved regulatory mechanism of gene expression in which introns are excised and exons are ligated in different combinations to create various alternative mRNA isoforms (Park et al., 2018). It can be categorized into seven simple patterns: 1) alternative 5′ splice site (A5SS); 2) alternative 3′ splice site (A3SS); 3) skipping exon (SE); 4) retained intron (RI); 5) mutually exclusive exons (MXE); 6) alternative first exon (AFE); and 7) alternative last exon (ALE) (Park et al., 2018). Alternative splicing is a major driver of the diversity of transcriptome and proteome in higher eukaryotic organisms and plays a vital role in cell differentiation, proliferation, apoptosis, organ development, and the genesis of human disease. (Xiong et al., 2015; Scotti and Swanson, 2016; Xing et al., 2020). Alternative splicing is also essential for mammalian early embryogenesis to generate a viable organism from a fertilized cell. Xing et al. (2020) constructed alternative splicing atlas and analyzed their functions during preimplantation in mouse. Wang et al. (2022) illustrated the function of alternative splicing during hematopoietic stem cell formation. Shen et al. (2021) reported that spliceosomal repression in mouse ESCs drives a pluripotent-to-totipotent state transition. The role of alternative splicing in sex determination of vertebrates has been intensively investigated (Gómez-Redondo et al., 2021). The alternative polyadenylation during zebrafish development was extensively identified (Li et al., 2012; Ulitsky et al., 2012). Nevertheless, much less is known about the detailed temporal and spatial patterns and regulatory role of alternative splicing during embryogenesis of zebrafish.

In this work, we utilized time-series RNA-seq dataset across 18 time points from one cell to 5 days post-fertilization to dissect the alternative splicing dynamics during embryo development of zebrafish by bioinformatics pipeline. The alternative splicing atlas was constructed in 18 time-series development stages and 2,412 differential alternative splicing (DAS) events embedded in 2,106 genes were identified between the consecutive development stages. Then, we detected isoforms switch (IS) events during embryogenesis and inferred IS event is implicated in embryogenesis. The inclusion level of isoform is the most important index of alternative splicing events. It was shown that clustering of biological replicates only based on the inclusion level of isoforms is comparable with that based on gene expression level. Besides, the present work also indicated that splicing factor (SF) is expressed in a developmental stage-specific manner, especially co-orthologous splicing factors. The dynamic features of AS and DAS events revealed that alternative splicing activation is synchronized with zygotic genome activation and alternative splicing is coupled with transcription during embryo development in zebrafish. This study is expected to be helpful for elucidating the molecular and cellular mechanisms of embryo development in zebrafish.

## 2 Materials and Methods

### 2.1 Construction of the Dataset

White et al. (2017) sequenced mRNA expression of 18 developmental stages of zebrafish on Illumina HiSeq 2,500 platform in 100 bp paired-end read mode with strand specificity and submitted the sequencing data to ENA database (Accession number: ERP014517). We downloaded and curated the transcriptome data of 90 samples with five biological replicates of each development stage (Supplementary Table S1). The 18 developmental stages are consisted of 1-cell, 2-cell, 128-cell, 1k-cell, dome, 50% epiboly, shield, 75% epiboly, 1–4 somites, 14–19 somites, 20–25 somites, prim-5, prim-15, prim-25, long pec, protruding mouth, post-developmental day 4, post-developmental day 5, which cover before and at the onset of zygotic transcription, gastrulation, somitogenesis, prim stages, and post-fertilization. Supplementary Table S1 showed that the average sequencing depth of the 90 samples is about 6.6 million. The data features of paired-end sequencing mode, over three biological replicates and the higher sequencing depth guaranteed the feasibility of downstream alternative splicing analysis.

FastQC (v0.11.8) (Andrews, 2010) and Trimmomatic (v.0.38) (Bolger et al., 2014) were used to perform quality control analysis of raw reads. FastQC analysis showed the quality of 5′ and 3′ end of reads is lower. Trimmomatic was used to remove low quality reads and cut 5 nt from 5′ and 3′ end of reads, respectively. All parameters were set to their default values with exception of read length value. All reads were outputted with a read length of 90 bp. The average surviving rate and sequencing depth after quality control are 86.0% and 5.7 million (Supplementary Table S1). Furthermore, we observed that 52.63% reads are mapped to exon-exon junctions, which enabled the reliable determination of the splice isoforms.

### 2.2 Transcript and Gene Expression Quantification

Transcript quantification was performed using the Salmon (v0.12.0) tool developed by Patro et al. (2017). Salmon is an RNA-seq data analysis tool for rapid quantification of transcripts without sequence alignment, mainly describing the expression of transcripts in terms of TPM (transcripts per million) values. The quantification process includes indexing based on transcriptome sequence file and transcript quantification based on sequencing files. The zebrafish annotation file (Danio_rerio.GRCz11.101.chr.gtf) and the matched reference transcriptome sequence file (Danio_rerio.GRCz11.cdna.all.fa) were derived from the Ensembl database, which contains 32,057 genes and 59,290 transcripts.

Under quasi-mapping mode, we used the reference transcriptome to build the index. Owing to the read length is greater than 75 bp, the hash parameter *k*-mer was set to 31. For quantification, library type selection parameter was set to A, the gcBias parameter was turned on for GC content bias calibration, and other parameters were default settings. TPM values in all samples were calculated for 50,794 transcripts corresponding to all coding genes. The output files of the quantitative analysis of transcripts for each sample were used as the input files of the tximport tool (R package, v1.14.2) and only the first of the overlapped genes was considered for tximport processing (Soneson et al., 2015). Finally, the transcript TPM matrix with 50,670 (transcripts) * 90 (samples) and the gene count matrix with 25,084 (genes) * 90 (samples) were constructed. This work mainly focused on coding genes and transcripts located on 25 chromosomes and all downstream analysis will aim at 25,084 coding genes extracted from annotation files.

### 2.3 Detection of Differentially Expressed Genes

The identification of differentially expressed genes (DEGs) during different developmental stages in zebrafish was performed using the DESeq2 tool (v1.26.0) based on the negative binomial distribution model (Love et al., 2014). The basic steps and parameters setting of DESeq2 can be summarized as follows: step 1: input the obtained gene expression matrix (row names are sample names and column names are gene names); step 2: set the grouping information and construct the dds object; step 3: filter out low-expressed genes: keep 24,005 genes expressed in at least five samples; step 4: the utilization of DESeq function: estimate size factors and dispersions, and fit model and test; step 5: set the threshold value and output results: DEGs with an adjusted *p* value < 0.05 and |fold change| ≥ 2 were accepted as significant.

### 2.4 The Analysis of Alternative Splicing Events

#### 2.4.1 The Detection of Alternative Splicing Events

Despite there being many detection approaches and quality alternative splicing events from RNA-seq, SUPPA2 exhibits excellent performance in terms of computing capacity, sequencing depth requirements, multiple conditions analysis, and detecting accuracy (Trincado et al., 2018). Here, SUPPA2 (v2.3) was available at https://github.com/comprna/SUPPA and all alternative splicing events were identified. The zebrafish annotation file (Danio_rerio.GRCz11.101.chr.gtf) was used to define splicing events *via* the generateEvents function of SUPPA2. Then, the TPM values of transcripts were employed as inputted file of psiPerEvent function to compute the inclusion level (Percentage Spliced Inclusion, PSI) of every alternative splicing event. Next, differential splicing events analysis was performed using the diffSplice function to calculate the PSI change (ΔPSI) and *p* value across multiple development stages based on TPM values of transcripts. Criteria for determining differential alternative splicing events in contrast group is that 1) splicing change (|ΔPSI|) between two different developmental stages showed ≥0.1 . 2) ΔPSI differs significantly with p value<0.05 (Xing et al., 2020).

#### 2.4.2 Identification of Isoform Switch

Isoforms switch occur when the relative abundance of a pair of transcripts reverses under different conditions. We applied the TSIS R package to perform the isoforms switch analysis, which is a powerful tool to detect significant alternatively spliced isoforms switch in time-series transcriptome data (Guo et al., 2017). Significant isoforms switches were identified using the following filtering parameters: 1) the probability of switch was set to >0.5; 2) the sum of the average differences cutoff of the two isoforms >1; 3) *p*-value cutoff of significance difference before and after the switch <0.05; 4) min time in interval ≥2.

#### 2.4.3 The Collection of Splicing Factor

At present, there is no database of splicing factors in zebrafish. We, respectively, manually curated 509 and 455 splicing factors of human and mouse by screening literatures (Sebestyén et al., 2016; Seiler et al., 2018; Xing et al., 2020; Zhang et al., 2020; Shen et al., 2021) and databases (Giulietti et al., 2013). Then, a total of 428 unique splicing factors in zebrafish were collected by homology analysis with splicing factors in human and mouse, and covered many SR proteins and hnRNP proteins. The detailed information of splicing factors in zebrafish was listed in sheet1 of Supplementary Table S2. Furthermore, the *k*-means method-based gene expression was employed to analyze the time-course dynamics of splicing factors expression.

### 2.5 The Functional Enrichment Analysis

GO and KEGG enrichment analysis of DEGs, splicing factors, and genes undergoing differential alternative splicing were carried out by the ClusterProfiler (v4.1.4) package in R, which provides a user-friendly visual analysis tool (Yu et al., 2012). The zebrafish annotation information available on Bioconductor (org.Dr.eg.db, v3.12.0) was downloaded in the R environment for enrichment analysis. The statistical significance cutoffs for all GO and KEGG enrichment analysis were set to *p*. adjust < 0.05.

## 3 Results

### 3.1 The Overview of Gene Expression During Embryogenesis in Zebrafish

Alternative splicing plays a remarkably important role in increasing protein diversity by allowing one gene to generate distinct proteins and increasing the complexity of gene expression regulation. To study the function of alternative splicing in embryo development of zebrafish, we firstly investigated gene expression during the embryo development. Gene expression analysis of embryo development was implemented by bioinformatic method depending on RNA-seq data obtained from the study of White et al. (2017), consisting of 18 developmental stages throughout MZT, gastrulation, somitogenesis, pharyngula stages, and post-fertilization (see Materials and Methods). The transcripts and genes were quantified via free-alignment strategy. We only focused on 25,107 coding genes screened from the latest annotation files of zebrafish. For reducing the transcriptional noise and improving computational speed, we selected the protein-coding genes that were expressed in at least five or more samples for downstream analysis. Ultimately, the gene count matrix with 24,005 protein-coding genes along the rows and 90 samples along the column was created. All downstream analysis involved in gene expression was performed based on this matrix.

#### 3.1.1 The Dynamic Gene Expression in Embryo Development

**FIGURE 1:**
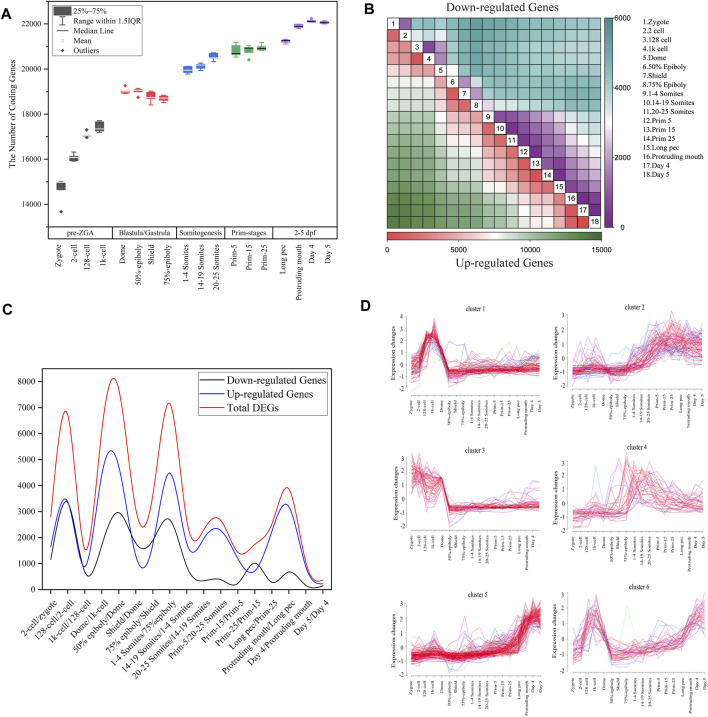
Gene expression dynamics during zebrafish development. **(A)** The boxplot of detected coding genes at 18 developmental stages. **(B)** The heatmap of DEGs between any two stages during embryogenesis. In all contract groups of DEGs, the late developmental stage is numerator, and the early developmental stage is denominator. The number of upregulated and downregulated genes are represented by the colors in the bottom and upper triangular, respectively. **(C)** The distribution of the number of differentially expressed genes for all consecutive stages of embryo development. The upregulated or downregulated gene refer to a gene that is upregulated or downregulated between two consecutive time points **(D)** Cluster analysis of downregulated genes between dome and 1k-cell stages based on Mufzz.

Developmental processes require precise spatio-temporal regulation of gene expression. We used time-series RNA-seq data to detect the dynamics of gene expression and identified 14,655, 16,075, 17,081, 17,429, 19,024, 19,004, 19,766, 18,695, 19,959, 20,125, 20,544, 20,811, 20,809, 20,947, 21,233, 21,901, 22,132, and 22,064 expressed genes (TPM > 0) in 18 development stages (Figure 1A). Besides, 10,070 protein-coding genes are expressed in all samples. In nearly all animals, maternally supplied RNAs and proteins regulate the initial events of embryogenesis while the zygotic genome remains transcriptionally silent. Transcriptional control is then passed to the zygote through the MZT process, during which the degradation of maternal products is orchestrated with ZGA (Schulz and Harrison, 2019). During MZT, ZGA is activated gradually. It is well known that ZGA in mouse is implemented by two transcriptional waves, of which a minor wave (minor ZGA) occurs at the mid-one-cell stage and a major wave is activated after 2-cell stage (major ZGA) (Abe et al., 2018). Some reports revealed that the minor wave of ZGA in zebrafish is initiated between the 64-cell and 256-cell stages (Aanes et al., 2011; Lee et al., 2013; Heyn et al., 2014; Wragg and Müller, 2016; Liu et al., 2018). However, we found the number of expressed genes is significantly elevated from zygote to 2-cell stages. Thus, we extrapolated that minor ZGA in zebrafish may be initiated at more earlier stage, such as zygote or 2-cell stage. By analyzing the expression profiles of 331 zygotically expressed genes extracted from Heyn et al. (2014), we further confirmed this extrapolation (Supplementary Figure S1). We also observed that major ZGA occurs at 1k-cell and dome stages of blastula. Due to the degradation of maternal mRNAs and the ending of major ZGA, the number of detected coding genes during shield and 75% epiboly stages of gastrulation is decreased. During somitogenesis and prim-stage, the cells are highly differentiated with organogenesis and lots of tissue-specific genes are activated. Therefore, the number of detected coding genes is slightly increased in these stages.

To more clearly elucidate the dynamic nature of the gene expression during zebrafish development, the DESeq2 package with padj < 0.05 and |fold change| ≥ 2 was employed to identify DEGs between each any two stages of embryo development (Figure 1B; Supplementary Table S3). The results showed that the number of up-regulated genes is significantly greater than that of downregulated genes. We can deduce that the number of zygotic genes is far more than maternal genes. By dissecting the DEGs from 18 consecutive stages of embryo development, peaks of the upregulated genes between 128-cell and 2cell stages, dome and 1k-cell stages, 1–4 somites and 75% epibody stages, prim-5 and 20–25 somites, long pec and protruding mouth stages were observed (Figure 1C). It suggested that in the process of MZT, the late period of gastrulation to somitogenesis and the pharyngula period are finished with lots of gene activation and upregulation of gene expression. By comparing the distribution feature of up-regulated and downregulated genes, it was also noted that the activation of zygotic genes simultaneously occurs with degradation of maternal RNA from 1-cell stage. The peaks of downregulated genes are overlapped with upregulated genes until 1–4 somites stage where the majority maternal RNAs has been degraded.

Furthermore, we employed Mufzz method to detect time-series expression profiling of DEGs in embryonic development. For example, the 1,706 downregulated genes between dome and 1k-cell were classed into six clusters (Figure 1D). Cluster 1 expression levels increase from the zygote stage, reach the peak at the 1k-cell and decrease sharply after 1k-cell stage, and then keep the lower expression level until day 5 stage. Cluster 3 expression levels reach the peak at the zygote and 2-cell stages and decrease until 50% epiboly, and then keep the lower expression level until day 5 stage. Cluster 4 expression levels slightly decrease at dome stage and steadily risen during gastrulation and somitogenesis, and then slowly decrease. Other clusters expression levels show an increasing trend after dome stage. These results indicated that the downregulated DEGs between dome and 1k-cell may become re-expressed later during development. We also employed Mufzz method to perform cluster analysis of other DEGs between neighboring stages (Supplementary Figure S2). It further implied the DEGs expression is dynamic during embryo development.

By analyzing the DEGs between zygotes and other 17 stages of embryo development, we also extrapolated the activation of zygotic genes and degradation of maternal genes are performed step by step. Besides, it was showed that the number of DEGs between day 4 and day 5 after fertilization was the smallest, indicating that the oscillation of transcriptome in small early juvenile is slight.

#### 3.1.2 The Stage-Specific Expression of Splicing Factors During Embryogenesis

Alternative splicing is precisely regulated by the combination of *cis*-regulatory sequences, *trans*-acting splicing factors, chromatin organization, histone modification, DNA metyhylation, RNAPII elongation, etc., of which splicing factors bind to *cis*-elements and promote or inhibit splice site recognition (Xing et al., 2016). In recent years, it was revealed that variable expression of splicing factors in different cell types, developmental stages and tissues or at different external conditions in mouse or human mediates differential recognition of exons, thus producing alternative mRNA isoforms (Shenasa and Hertel, 2019). Splicing factor NOVA2 regulates brain development by mediating the alternative splicing of *DAB1*. During heart development, splicing factor RBM20 regulates the alternative splicing of *titin* and RBM20 mutations will cause cardiomyopathies in human (Baralle and Giudice, 2017). The splicing factors ZRSR1 or ZRSR2 are necessary for ZGA and are essential for the conversion of induced pluripotent stem cells into 2C-like cells in mouse (Gómez-Redondo et al., 2020). The splicing factor RBFOX2 plays a role in placental development (Goldman-Wohl et al., 2020). The splicing factors CPSF3, hnRNP UL1, and TIA1 can repress embryonic fibroblasts reprogramming in mouse (Vivori et al., 2021).

**FIGURE 2:**
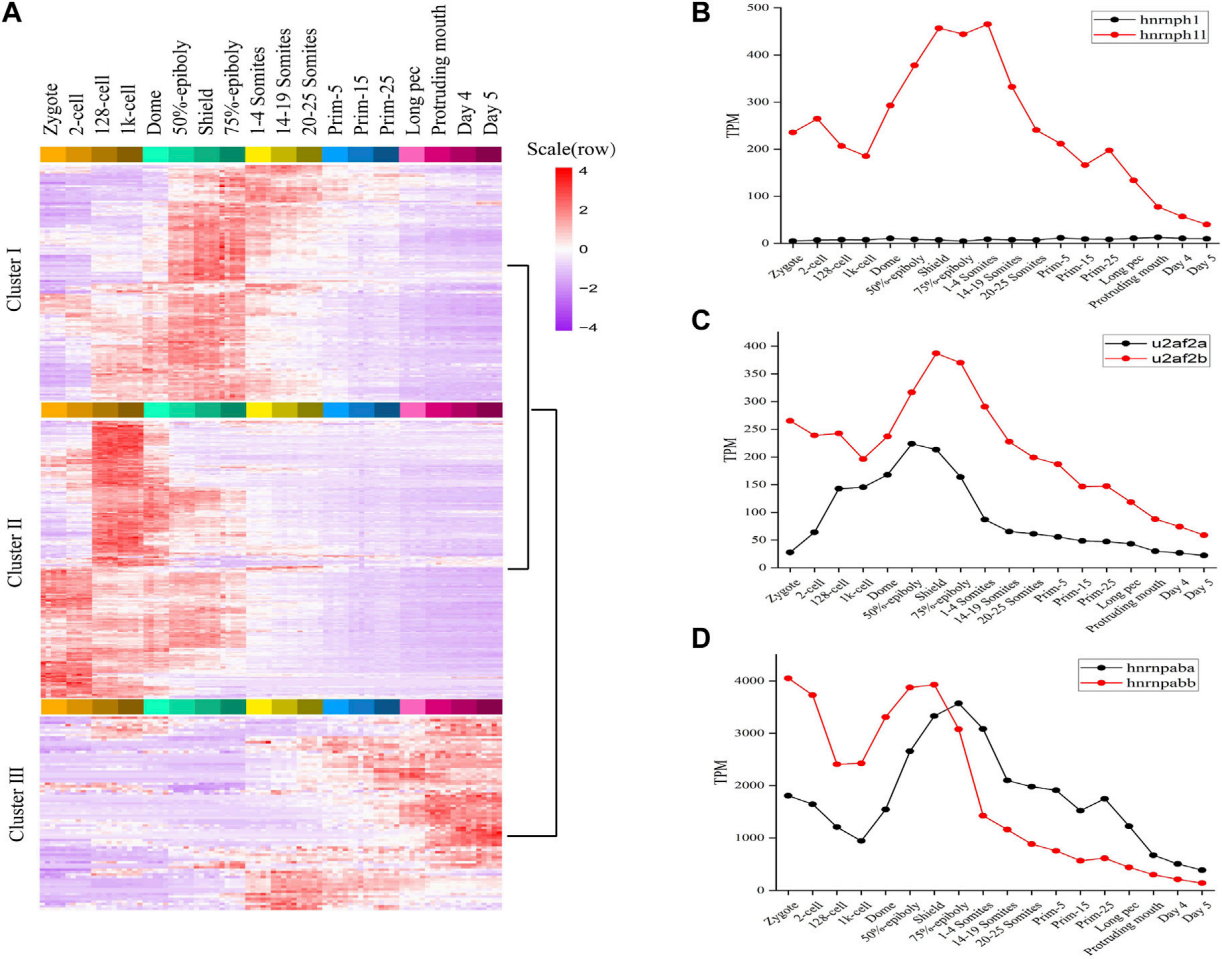
The expression profiles of splicing factors in zebrafish. **(A)** The expression map and unsupervised clustering of splicing factors during embryogenesis development. **(B–D)** The expression level of three categories splicing factors having two orthologous forms. The expression level of hnrnph1/hnrnph1l, u2af2a/u2af2b and hnrnpaba/hnrnpabb was plotted in **(B–D)**, respectively.

Nevertheless, whether the splicing factors expression is stage-specific and what’s the function of splicing factors during embryogenesis in zebrafish are unclear. Due to lack of splicing factors database of zebrafish, we constructed splicing factors database in zebrafish based on literature mining and homology analysis (Section 2.4.3). This dataset is consisted of 428 unique splicing factors in zebrafish and covers many SR proteins (rp54, srsf1a, srsf9, and tra2a) and hnRNP proteins (hnrnpaba, hnrnpabb, hnrnpd, hnrpdl, hnrnph1, hnrnph1l, hnrpl, hnrnpub, hnrnpua, hnrnpul1, hnrnpul1l, rbmx, ptbp1b, syncrip, and fmr1). To examine specificity of splicing factors along embryogenesis, we employed the *k*-means method to perform the specific analysis of splicing factors expression throughout 18 developmental time points. The splicing factors were divided into three clusters, of which 210 splicing factors were classified as cluster 1, 139 splicing factors were classified as cluster 2, 79 splicing factors were classified as cluster 3 (Figure 2A). Splicing factors in cluster 1, 2, and 3 are mainly expressed among gastrulation, during MZT and after gastrulation, respectively. Furthermore, cluster 1 and cluster 2 can be joined into a cluster, implying the expression profiles of splicing factors in early stages of embryogenesis is more similar compared with late stages of embryogenesis. GO enrichment showed that these splicing factors were highly related to splicesome assembly, mRNA metabolic process, mRNA splicing, etc. (Supplementary Figure S3). Remarkably, except for splicesome and mRNA surveillance pathway, the splicing factors in cluster 1 were also significantly enriched in RNA degradation (Supplementary Figure S4). It suggested the maternal RNAs are vastly degraded among gastrulation, which explains why there is a valley of detected coding genes during gastrulation in Figure 1A.

Like many teleost fish, zebrafish are believed to have experienced an additional genome wide duplication event. As a result, they often have two co-orthologs in contrast to a single copy gene in human and other mammals (Taylor et al., 2003). We found 24 splicing factors in human or mouse having two co-orthologs in zebrafish. The detailed information of 24 pairs orthologous splicing factors was listed in sheet2 of Supplementary Table S2. It covers hnRNP proteins, SRPK proteins, etc. The expression profiles of 24 co-orthologs during 18 stages of zebrafish development (Supplementary Figure S5) were characterized and some very interesting and meaningful results were gained.

Firstly, 24 co-orthologs of splicing factors can be classified into three categories based on the expression profiles. Category 1: One orthologue of splicing factor is dynamically expressed and another is expressed with very lower level during 18 consecutive development stages, such as hnrnph1/hnrnph1l, jupa/jupb, rnf213a/rnf213b (Figure 2B; Supplementary Figure S5). Category 2: The co-orthologs of splicing factor are expressed simultaneously during embryo development, but one of the orthologue is dominant, another is expressed with lower level, such as hnrnpua/hnrnpub, hnrnpc/zgc:55,733, ptbp1a/ptbp1b, celf3a/celf3b, eef1a1a/eef1a1b, msi2a/msi2b, nhp2l1a/nhp2l1b, rbpms2a/rbpms2b, rnf34a/rnf34b, rps26/rps26l, and u2af2a/u2af2b (Figure 2C; Supplementary Figure S5). Category 3: The co-orthologs of splicing factor are expressed simultaneously, and the dominant contributor is switched between co-orthologs during embryo development, such as hnrnpaba/hnrnpabb, hnrnpul1/hnrnpul1l, cnot4a/cnot4b, ivns1abpa/ivns1abpb, nova1/nova2, ppp1caa/ppp1cab, rbfox3/rbfox3l, rbm4.1/rbm4.2, rbms2a/rbms2b, and srpk1a/srpk1b (Figure 2D; Supplementary Figure S5). These results revealed that the expression profiles of two co-orthologs of splicing factor are different during embryo development and the functions of the two co-orthologs are diverging.

Secondly, hnrnpaba/hnrnpabb, hnrnph1l, hnrnpua/hnrnpub, hnrnpul1/hnrnpul1l, hnrnpc/zgc:55,733, and ptbp1a/ptbp1b, which, respectively, are co-orthologs of splicing factor HNRNPAB, HNRNPH1, HNRNPU, HNRNPUL1, HNRNPCL3, and PTBP1 in human and mouse, are expressed with high level in early developmental stages, especially hnrnpaba/hnrnpabb (Supplementary Figure S5). It confirmed that alternative splicing is regulated by the activities of hnrnp protein family in zebrafish, especially in early developmental stages.

Moreover, the expression profiles of all co-orthologs are dynamic and stage-specific (Supplementary Figure S5). The co-orthologs including cnot4a/cnot4b, ivns1abpa/ivns1abpb, msi2a/msi2b, rnf34a/rnf34b, rbpms2a/rbpms2b, and rnf213a/rnf213b are highly expressed during ZGA. The co-orthologs including celf3a/celf3b, eef1a1a/eef1a1b, nova1/nova2, rbfox3/rbfox3l, and rps26/rps26l are highly expressed after somitogenesis. The co-orthologs including hnrnpaba/hnrnpabb, hnrnph1/hnrnph1l, hnrnpua/hnrnpub, hnrnpul1/hnrnpul1l, jupa/jupb, nhp2l1a/nhp2l1b, ptbp1a/ptbp1b, rbms2a/rbms2b, rbm4.1/rbm4.2, and u2af2a/u2af2b are highly expressed between gastrulation and somitogenesis. The co-orthologs including hnrnpc/zgc:55733, ppp1caa/ppp1cab, and srpk1a/srpk1b are highly expressed before ZGA, and we inferred that these factors should be maternal genes.

Taken together, these results revealed that splicing factors expression are stage-specific during embryonic development of zebrafish and is abundantly expressed in the process of MZT. We inferred that many splicing factors are activated with ZGA and play critical roles during MZT. It indicated that alternative splicing is activated along with ZGA and play a vital role in embryogenesis mediated by splicing factors.

### 3.2 Dynamic Changes of Alternative Splicing During Embryogenesis

Alternative splicing is widespread and exerts critical functions during embryo development of mammals (Tian et al., 2020; Xing et al., 2020). However, the alternative splicing profile and function during embryogenesis of zebrafish haven not been revealed. Here, we systematically examined the dynamics and function of alternative splicing during zebrafish embryonic development.

#### 3.2.1 The Construction of an Alternative Splicing Atlas in Embryogenesis Development

**FIGURE 3:**
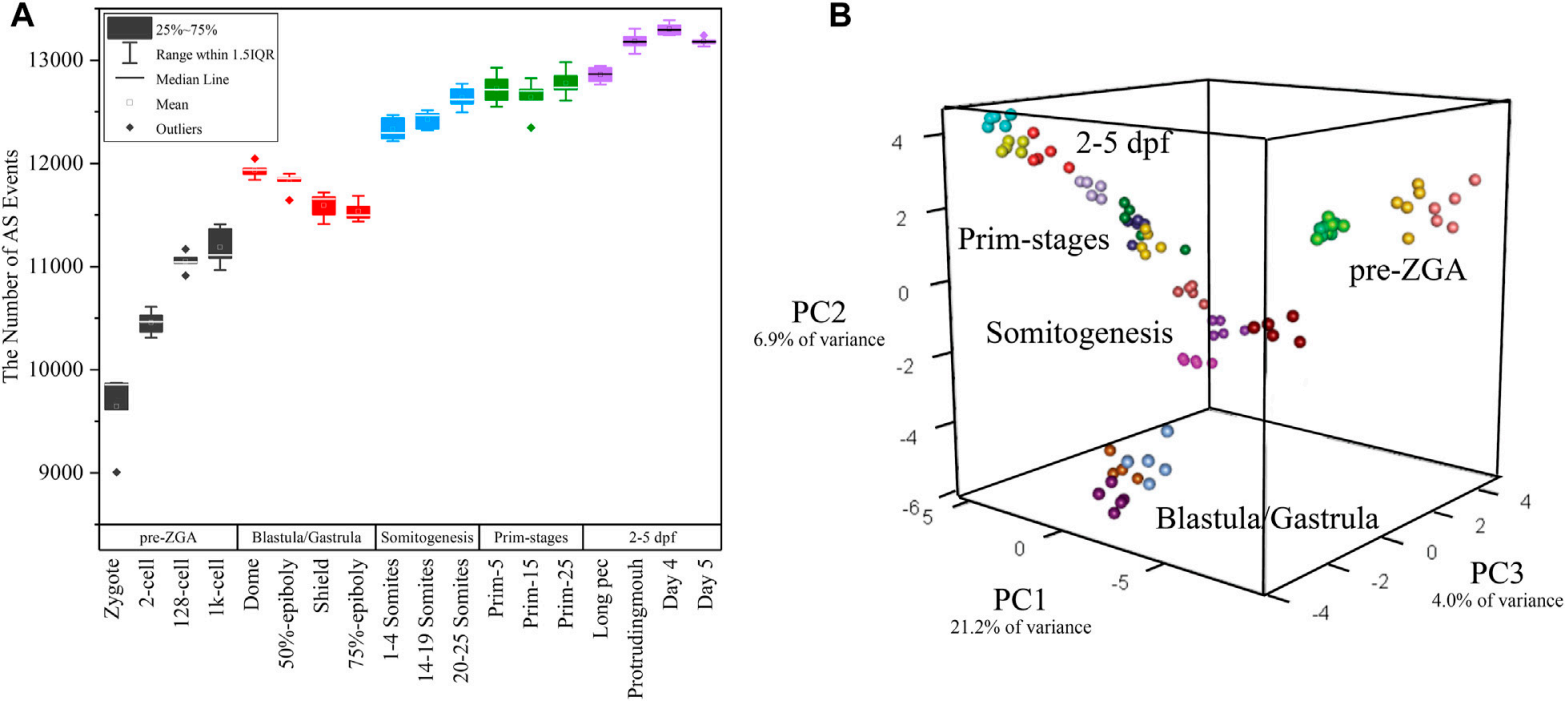
The distribution of alternative splicing events in 18 developmental stages. **(A)** The boxplot of detected alternative splicing events at 18 developmental stages. **(B)** 3D plot of PCA during embryogenesis stages. The samples in different stages were denoted with different colors.

We employed the SUPPA2 tool to examine alternative splicing dynamics in 18 developmental stages of zebrafish. Firstly, we, respectively, generated 2,184, 2,688, 3,943, 1756, 302, 3,937, and 817 alternative splicing events matching to A5SS, A3SS, SE, RI, MXE, AFE, and ALE based on annotation file (Table 1). The 15,629 alternative splicing events are embedded in 7,746 coding genes. The ratio of coding genes undergoing alternative splicing is 30.88%, which is remarkably lower than that in human (76.67%) and mouse annotation file (52.05%). Similar to mouse genome, the proportions of SE and AFE are highest, which account for 25.23% and 25.19% of all alternative splicing events, respectively. Besides, the proportion of RI events is 11.24%, which is elevated in comparison with mouse genome. Then, the PSI values denoting inclusion level of alternative splicing events in 90 samples throughout 18 developmental stages were calculated (Figure 3A). For reducing false positive of alternative splicing events in every developmental stage, only the events in which the PSI value is in the range of 0–1 in every replicate sample were retained. The distribution features of the number of total AS events and every AS pattern along 18 developmental stages were depicted (Supplementary Figure S6). It was showed that the highest number of alternative splicing events (12,051) presented in day 4 stage and the lowest number of alternative splicing events (7,744) presented in zygote stage. It was indicated that the number of alternative splicing events is increased with embryogenesis, especially from zygote to dome, which is coincided with the tendency of the number of coding genes (Figure 1A). We also normalized the number of alternative splicing events and alternative spliced genes to the number of expressed genes at each stage. The results showed that the profiles of normalized number of alternative splicing events and alternative spliced genes are similar, and more alternative splicing activity should still be observed during ZGA. It suggested that the activating of alternative splicing is synchronized with ZGA and is finished step by step. Moreover, the alternative splicing number remarkably decreases during gastrulation, which may be related with the degradation of maternal transcripts.

**TABLE 1:** Frequency of alternative splicing events based on genome annotation information in zebrafish, human, and mouse.

	A5SS	A3SS	SE	RI	MXE	AFE	ALE	SUM
Mouse	6335	7,089	13,945	3,059	1203	23,054	3,912	58,597
	**10.81%**	**12.10%**	**23.80%**	**5.22%**	**2.05%**	**39.34%**	**6.68%**	**100%**
Human	13,328	14,267	35,177	5675	4467	71,015	15,765	159,694
	**8.35%**	**8.93%**	**22.03%**	**3.55%**	**2.80%**	**44.47%**	**9.87%**	**100%**
Zebrafish	2,184	2,688	3,943	1756	302	3,937	817	15,627
	**13.98%**	**17.20%**	**25.23%**	**11.24%**	**1.93%**	**25.19%**	**5.23%**	**100%**

The bold number denotes the percentage of different alternative splicing patterns against the sum of alternative splicing events.

White et al. (2017) performed principal component analysis (PCA) based on gene expression. Here, PCA was again performed only based on the PSI value of alternative splicing events in every sample. PCA showed the first three principal components (PCs) explained over 30% of the variance in the data (Figure 3B) and the first component accounts for 21.2% of the observed variation. Surprisingly, it was showed that the clustering result is exactly similar with that in the work of White et al. (2017). The biological replicates from the same stage can be clustered together and the transition from one to another stage was identical with actual developmental time (Figure 3B). Thus, these 90 samples have a high reliability and feasibility for downstream analysis. It supported the statement that alternative splicing is stage-specific during early embryogenesis and PSI value can be used as a prominent index of PCA.

#### 3.2.2 The Differential Alternative Splicing Events Are Crucial for Embryogenesis

**FIGURE 4:**
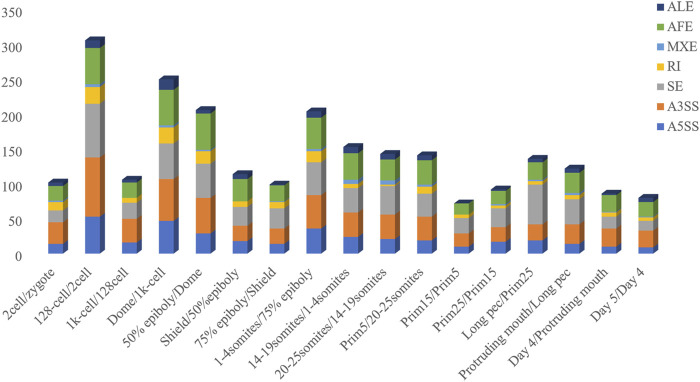
Frequency of differential alternative splicing events between consecutive developmental stages.

**FIGURE 5:**
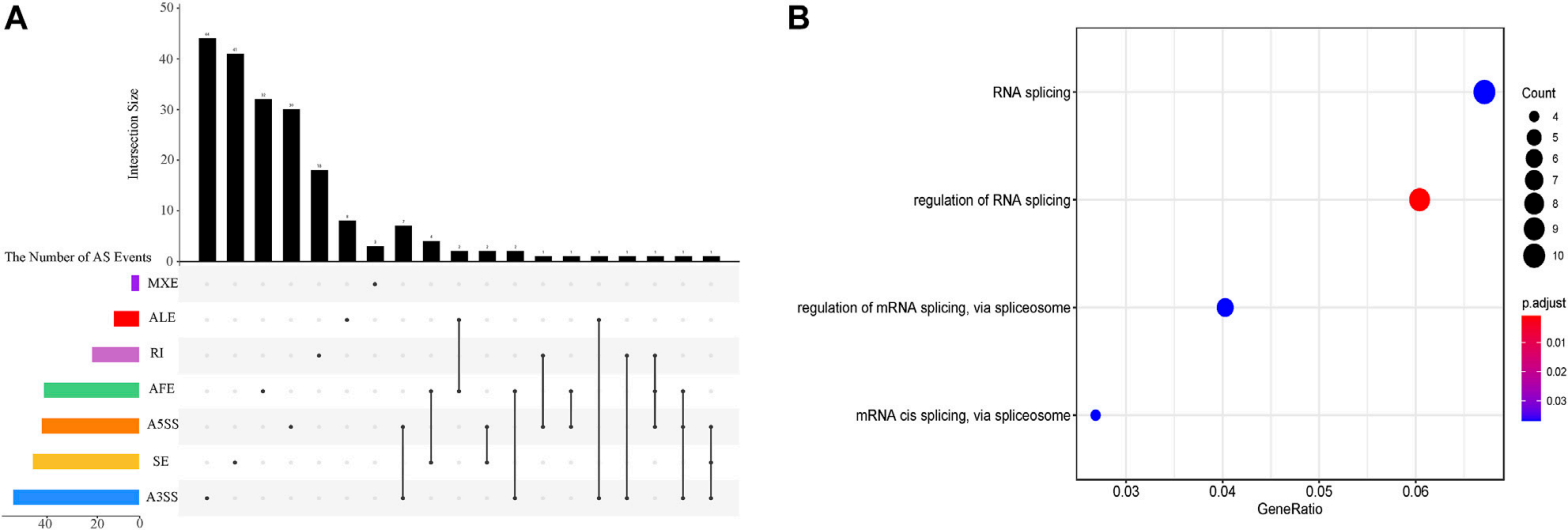
The characterization of differential alternative splicing events between dome and 1k-cell. **(A)** Upset plot depicts the interactions among the seven patterns of alternative splicing events between dome and 1k-cell. **(B)** GO functional enrichment of differentially alternatively spliced genes between dome and 1k-cell.

The inclusion level of transcript isoform is dynamic and play essential regulatory roles during embryo development and disease occurrences (Baralle and Giudice, 2017; Xing et al., 2020). In this work, the diffSplice module of SUPPA2 with the threshold of |ΔPSI| ≥ 0.1 and *p*-value < 0.05 was used to identify differential alternative splicing events (DAS) between any two time points of 18 developmental stages (Supplementary Table S4). Of which, a total of 2,412 differential alternative splicing events derived from 2,106 differentially alternatively spliced genes (DASGs) were identified between the consecutive stages (Figure 4). It revealed differential alternative splicing is important in MZT and gastrulation. The proportions of A5SS, A3SS, SE, RI, MXE, AF, and AL in differential alternative splicing events are 15.34%, 24.79%, 24.95%, 6.88%, 1.62%, 22.18%, and 4.23%, respectively. It can be observed the A3SS, SE, and AF patterns account for the large proportions in the differential alternative splicing events. Obviously, the number of differential alternative splicing events during ZGA and gastrulation is greater, and the number is decreased during late embryogenesis. For example, the greater number of differential alternative splicing events was detected in 128-cell/2cell(306) and dome/1k-cell(250); the smaller number of differential alternative splicing events was detected in prim15/prim5(72) and day 5/day 4(80). The dynamic features of differential alternative splicing events implied that differential alternative splicing events may carry out important functions in the processes of MZT and gastrulation. Meanwhile, by overlap analysis of seven differential alternative splicing patterns and related genes between dome and 1k-cell, we found that most of the genes only include a differential alternative splicing event and a small amount of gene comprise two or three differential alternative splicing events (Figure 5A). This result suggested that the regulation of differential alternative splicing event during ZGA is occasionally performed through crosstalk of multiple alternative splicing events. Moreover, we also counted the differential alternative splicing events occurred in 428 splicing factors during early development in zebrafish. A total of 64 DAS derived from 29 unique splicing factors were identified during eight consecutive development stages from zygote to shield stage (Supplementary Figure S7; Supplementary Table S5). This demonstrated that some factors may regulate alternative pre-mRNA splicing by alternative splicing of themselves during embryogenesis development.

To further elucidate the biological functions of genes undergoing differential splicing events, we used clusterProfiler to perform GO functional enrichment analysis of differentially alternatively spliced genes between consecutive developmental stages (Supplementary Figure S8). It is surprising that a total of 29 differentially alternatively spliced genes between dome and 1k-cell are significantly enriched in four GO-terms of biological processes associated with RNA splicing regulation *via* splicesome (Figure 5B). Nevertheless, the GO terms of enrichment analysis between other consecutive developmental stages are not closely related with splicing regulation. The enrichment of these terms demonstrated that 1) there is more alternative splicing activity between 1k-cell and dome stages; 2) many splicing factors may be produced and a machine of alternative splicing is assembled with ZGA. These results also unveiled that alternative splicing is implicated in embryogenesis in zebrafish.

The crosstalk between gene expression and alternative splicing is important to reveal the AS dynamics during developmental stages. We took the dominant SE pattern in differential alternative splicing events between dome and 1k-cell as an example to investigate the correlation between DAS events and DEGs. A total of 51 differential SE events derived from 48 differentially alternatively spliced genes were identified. We gained the ΔPSI value of every differential SE event and ΔTPM value of DASGs corresponding every differential SE event between dome and 1k-cell. Then, the Pearson’s correlation coefficient was calculated (Supplementary Figure S9). A weak positive correlation between DAS and DEGs was showed. That is to say, when the Δ*PSI* value of DAS is elevated between two stages, the Δ*TPM* value of DASGs is tend to increase.

### 3.3 The Alternatively Spliced Isoforms Switch Analysis in Consecutive Developmental Stages of Embryogenesis

Most studies on early embryo development focused on gene expression regulation. Whereas, due to cotranscriptionally of alternative splicing in metazoan, alternative splicing plays important roles in embryogenesis. It must be emphasized that isoforms abundance differences generated by alternative splicing can’t be exposed by gene-level measurement. Thus, we investigated the isoforms switch events by TSIS program where the expression level of gene is constant, but the abundances of isoforms are reversed during development (Guo et al., 2017). As input file of TSIS, the abundance (TPM) of 7,505 transcripts involved with all differential alternative splicing events were extracted from transcript expression matrix. A total of 1864 significant (*p* < 0.05) isoforms switch events comprised of two transcript isoforms were identified in 2,106 unique differentially alternatively spliced genes.

**FIGURE 6:**
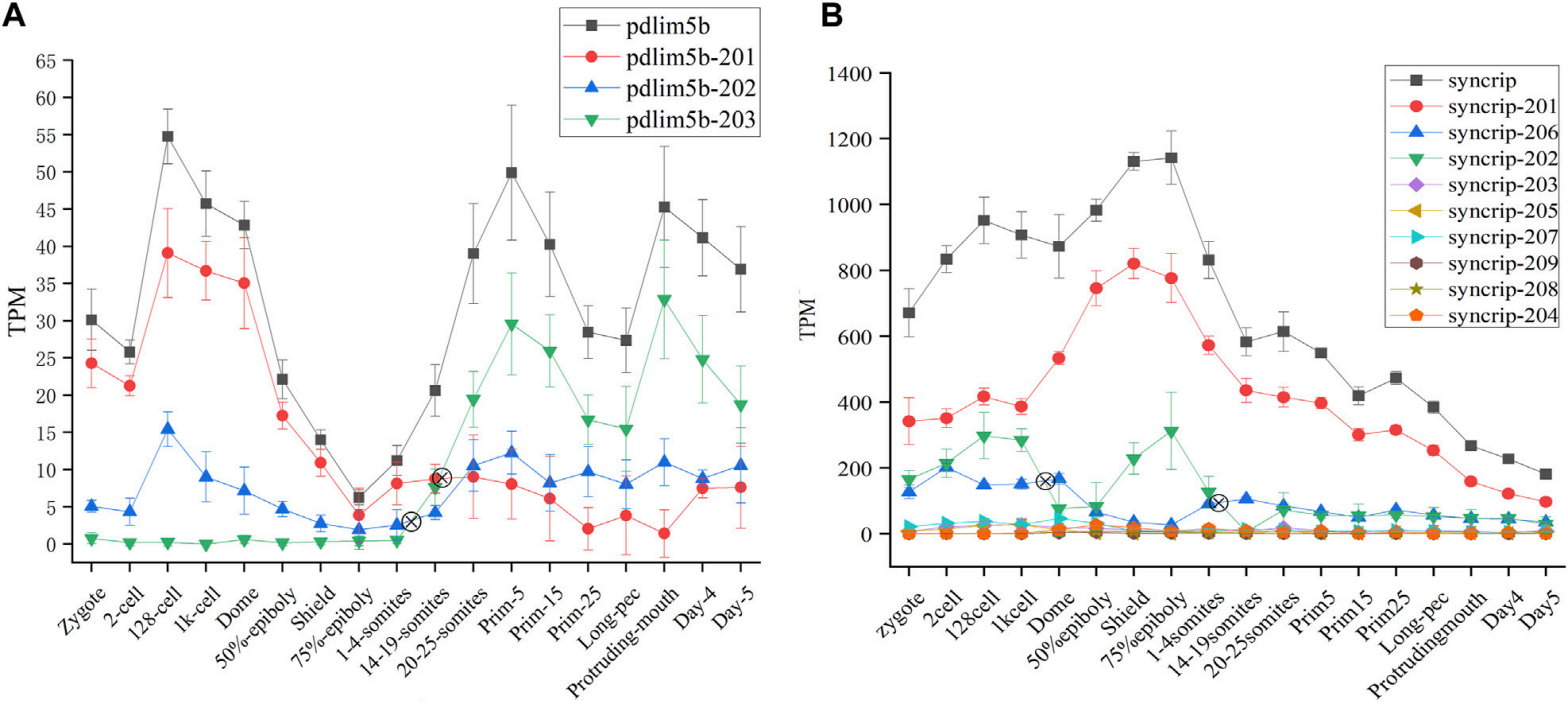
Expression profiles of *pdlim5b* **(A)** and *syncrip* genes **(B)** during 18 time-course stages. The *y*-axis denotes TPM of transcript isoforms. The symbol denotes switch point. The black line denotes gene expression level and other color lines denote transcript expression levels.

The protein product encoded by *pdlim5b* (*ENSDARG00000027600*) belongs to PDLIM protein family (PDZ AND LIM DOMAIN PROTEIN), and is implicated with heart and muscle development, actin cytoskeleton organization*.* The *pdlim5b* generates three different transcript isoforms, of which *pdlim5b*-201 and *pdlim5b*-203 are translated into the same protein made up of 628 residues with different 3′UTR, and protein product of *pdlim5b*-202 is consist of 197 residues. The alternative splicing atlas showed that the MXE event occurs between *pdlim5b*-201/*pdlim5b*-203 and *pdlim5b*-202. The transcript abundance can be modulated by MXE event in *pdlim5b*. The isoforms switch analysis indicated that the transcript abundances of three isoforms are dynamic during development, especially for *pdlim5b*-201 and *pdlim5b*-203 (Figure 6A). Although the protein product is same, the expression level of *pdlim5b*-201 is remarkably higher than that of *pdlim5b*-203 during ZGA process, and this tendency is reversed after gastrulation. It can be inferred that the reverse of *pdlim5b*-201 and *pdlim5b*-203 is involved in embryo development and may be related with 3′UTR regulation. Comparing to *pdlim5b*-201 and *pdlim5b*-203, the prominent feature of *pdlim5b*-202 is that the protein product lacks the C-terminal zinc finger domain. Thus, *pdlim5b*-202 may modulate embryo development *via* time-course–specific expression with different regulatory pathways.

*Syncrip*(synaptotagmin-binding, cytoplasmic RNA-interacting protein, *ENSDARG00000040184*) is a homologous gene of splicing factor *hnRNPQ* in human and mouse, which was included in splicing factor dataset of zebrafish (see sheet1 of Supplementary Table S2). This gene belongs to the subfamily of ubiquitously expressed heterogeneous nuclear ribonucleoproteins (hnRNPs). HnRNPs regulate alternative splicing, mRNA metabolism, and transport. Besides, *syncrip* is evolutionarily conserved across eukaryotes and participates in the regulation of neuronal and muscular development (Chen et al., 2020). Nine spliced transcript variants involved in A5SS, A3SS, SE, and AFL have been observed for this gene in zebrafish. Of which, all transcripts other than syncrip-204 can be translated into proteins with different amino acid residue, and the expression level of syncrip-201, syncrip-202, and syncrip-206 are higher, especially syncrip-206 (Figure 6B). We observed the dynamic expression profile of all transcripts during 18 developmental stages and detected remarkable isoforms switch events between syncrip-202 and syncrip-206. The switch of syncrip-202 and syncrip-206 results in the composition change of protein product of *syncrip*, in which the syncrip-202 and syncrip-206 are, respectively, translated into proteins with 632 and 225 amino acid residues. As splicing factor, the composition change of protein product of *syncrip* may result in the difference of regulatory target and function. Moreover, the embryo development during 18 time-point stages may be modulated by this mechanism.

## 4 Discussion

Alternative splicing occurs largely cotranscriptionally and is involved in embryogenesis, somatic cell reprogramming, and the transition from pluripotent ESCs to a totipotent state in mouse (Shen et al., 2021; Vivori et al., 2021). However, the study of alternative splicing during embryogenesis in zebrafish is poor. In this work, we profiled the gene expression and alternative splicing atlas of coding genes and investigated the function of alternative splicing in 18 developmental stages from zygote to 5 days post-fertilization. The results revealed the dynamic nature of alternative splicing during zebrafish embryo development.

Comparative analysis between the number of detected coding gene and alternative splicing events revealed the activation of alternative splicing is synchronized with ZGA. The previous studies also reported that alternative splicing and zygotic genes are activated simultaneously in mouse (Xing et al., 2020). With the ZGA, spliceosomes are produced and splicing machinery is assembled and activated. The coactivation of zygotic genes and alternative splicing during MZT process indicated co-transcriptional splicing maybe universal in zebrafish embryo development. The examination of expression profiles of splicing factors showed that the expression of splicing factors is stage-specific and many splicing factors were expressed during MZT process. The dynamic expression of splicing factors also indicated that alternative splicing events are diversified during embryo development. The analysis of co-orthologs of splicing factors indicated the expression profiles of two co-orthologs of splicing factor are different during embryo development and the functions of the two co-orthologs are diverging. This result provided a new insight into the regulation of alternative splicing from co-orthologs of splicing factors during embryo development in zebrafish. In future, we will attempt to conduct some experiments of splicing factor knockout or knockdown in zebrafish to elucidate the elaborate regulatory of splicing factor during embryo development.

**FIGURE 7:**
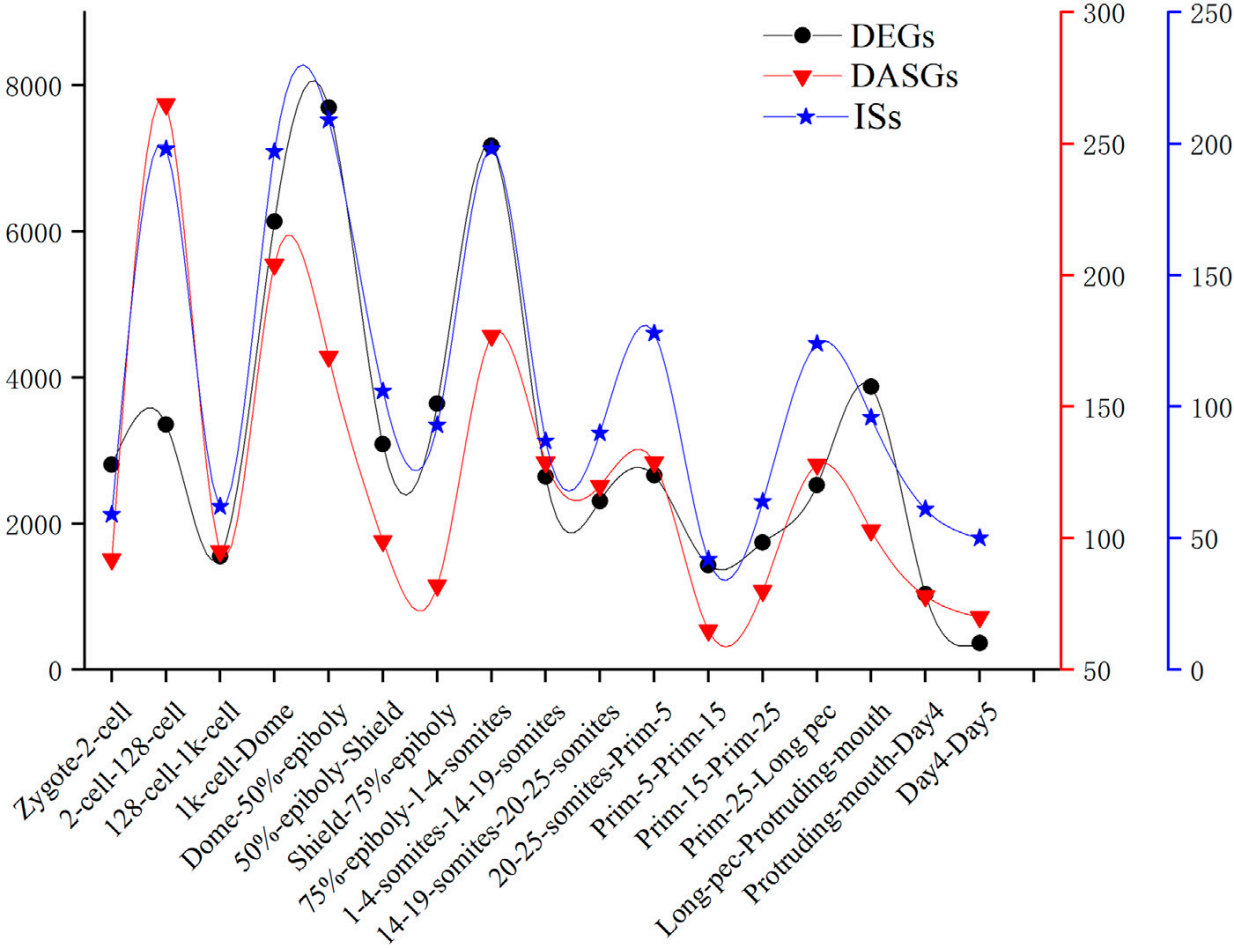
The number of DEGs, DASGs, and IS for every consecutive stages of embryo development. DASGs represent genes in which differential alternative splicing events was identified.

We detected the maximum alternative splicing events during the MZT process. Inclusion level (PSI value) is the most important index of alternative splicing events. We performed PCA analysis based on the PSI value of alternative splicing events and generated exactly similar result with that based on the level of gene expression. This result uncovered that inclusion level of alternative splicing events is a novel parameter of PCA analysis, which can be applied in cluster analysis and trajectory inference of development, etc. By identifying and analyzing differential alternative splicing events and differentially alternatively spliced genes, we found the number of differential alternative splicing events and differentially alternatively spliced genes during ZGA and gastrulation is greater. The numerous differential alternative splicing events during ZGA and gastrulation produce abundant proteins, which guaranteed ZGA and alternative splicing activation are finished successfully (Xing et al., 2020). The functional enrichment analysis demonstrated that lots of differentially alternatively spliced genes participate in metabolism of RNA splicing and alternative splicing regulation. It suggested that some genes may regulate alternative pre-mRNA splicing by alternative splicing of themselves during embryogenesis development. A positive correlation between DAS and DEGs was observed. It indicated that gene expression and alternative splicing are connected during developmental stages. The identification of 1864 significant IS provided a new insight to mine the regulated information derived from transcript isoforms, which always is hidden by gene expression. In addition, we conducted comparative analysis of the frequency of DEGs, DASGs, and IS (Figure 7). The result showed the peaks are overlapped and the crest values occur in MZT process, and once again elucidated the synchronization of activation of alternative splicing and ZGA.

Overall, our study revealed the extensive role of alternative splicing in regulating embryogenesis in zebrafish. By generating the alternative splicing atlas and identifying differential alternative splicing events analysis, we inferred that alternative splicing activation is synchronized with ZGA and discovered that alternative splicing is coupled with transcription during embryo development in zebrafish. Furthermore, we uncovered that splicing factors are expressed with stage-specificity. Also, we proposed that inclusion level of alternative splicing events can be used for cluster analysis as a novel parameter. This study will provide valuable guidelines for further experimental validations and computational analysis to elucidate the regulated mechanisms of embryotic development in zebrafish.

## Data Availability

The original contributions presented in the study are included in the article/Supplementary Material, further inquiries can be directed to the corresponding authors.

## Supplementary Material

Supplementary Figure S1 The expression profile of zygotically expressed genes during embryo development. A total of 592 zygotically expressed genes involved in coding and non-coding genes in zebrafish were provided by Heyn et al (2014). Given that only coding genes were analyzed in our work, a subset composed of 331 coding genes was used to plot the expression profile during 18 developmental stages in zebrafish.

Supplementary Figure S2 Cluster analysis of DEGs based on Mufzz.

Supplementary Figure S3 GO enrichment analysis of splicing factors.

Supplementary Figure S4 KEGG enrichment analysis of splicing factors.

Supplementary Figure S5 The expression level of splicing factors having two orthologous forms in zebrafish.

Supplementary Figure S6 The frequency distribution of all alternative splicing events and every alternative splicing pattern along 18 developmental stages.

Supplementary Figure S7 The frequency of differential alternative splicing derived from splicing factors during 8 consecutive development stages. The A5SS, A3SS, SE, RI, and AFE denote, respectively, 5 patterns of alternative splicing. DAS and DASG denote, respectively, differential alternative splicing events and differentially alternatively spliced genes.

Supplementary Figure S8 GO functional enrichment of differentially alternatively spliced genes between all consecutive time points of 18 developmental stages.

Supplementary Figure S9 The correlation between the ΔPSI of DAS and the ΔTPM value of DASGs.

Supplementary Table S1 The basic information of dataset.

Supplementary Table S2 Splicing factors of zebrafish. In sheet1, the gene names of all splicing factors were listed. In sheet2, splicing factors having two orthologous forms in zebrafish were exhibited. In sheet3, the TPM expression values of all splicing factors were provided.

Supplementary Table S3 The number of DEGs between any two developmental stages.

Supplementary Table S4 Frequency of differential alternative splicing events between any two points in 18 developmental stages. In sheet1, the total number of differential alternative splicing events between any two developmental stages was provided. In sheet2, the number of differential alternative splicing events of different AS patterns between any two developmental stages was provided.
