# Supplementary figures and images for: Temporal Dynamic Analysis of Alternative Splicing During Embryonic Development in Zebrafish
Source: Front Cell Dev Biol. 2022 Jul 8;10:879795. doi: 10.3389/fcell.2022.879795 (PMC9304896; doi:10.3389/fcell.2022.879795)

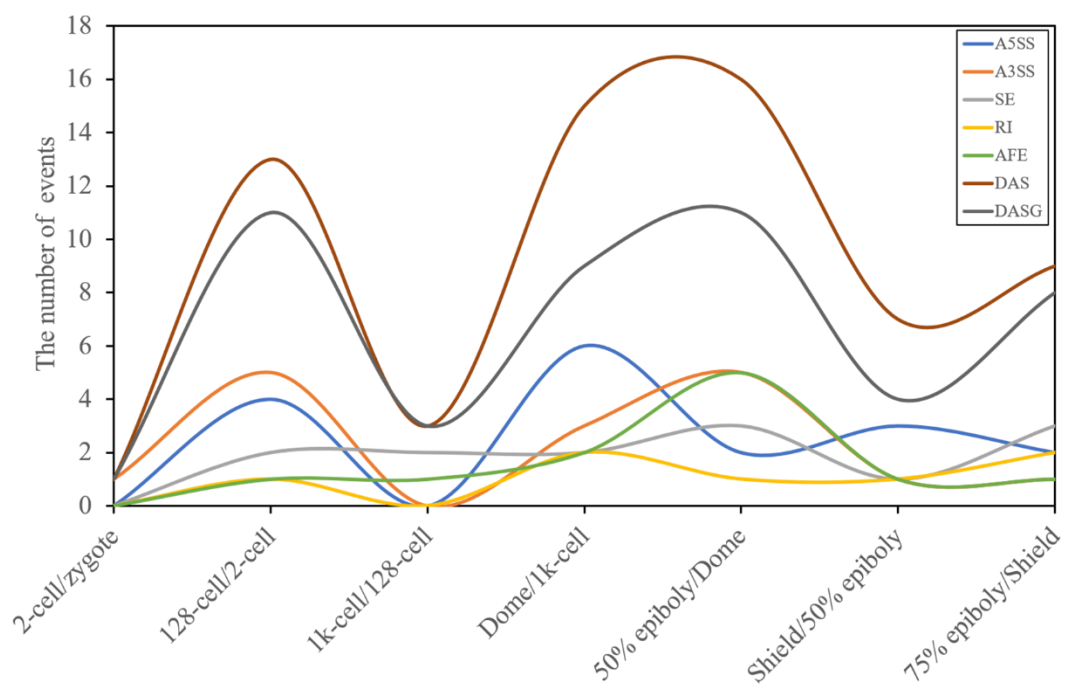

Supplement: Supplementary file 1 [file DataSheet7.PDF]

Cluster 1

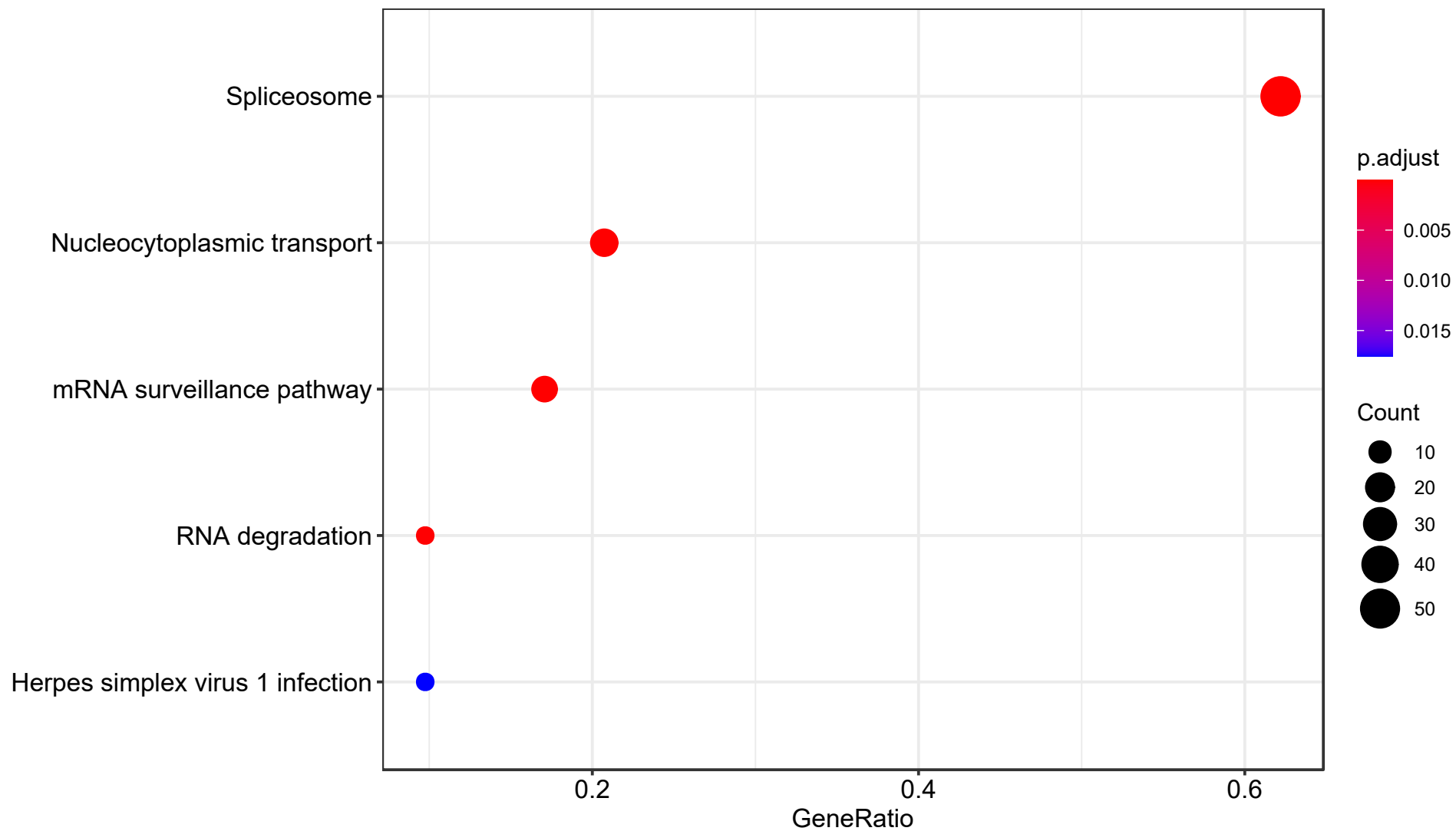

Cluster 2

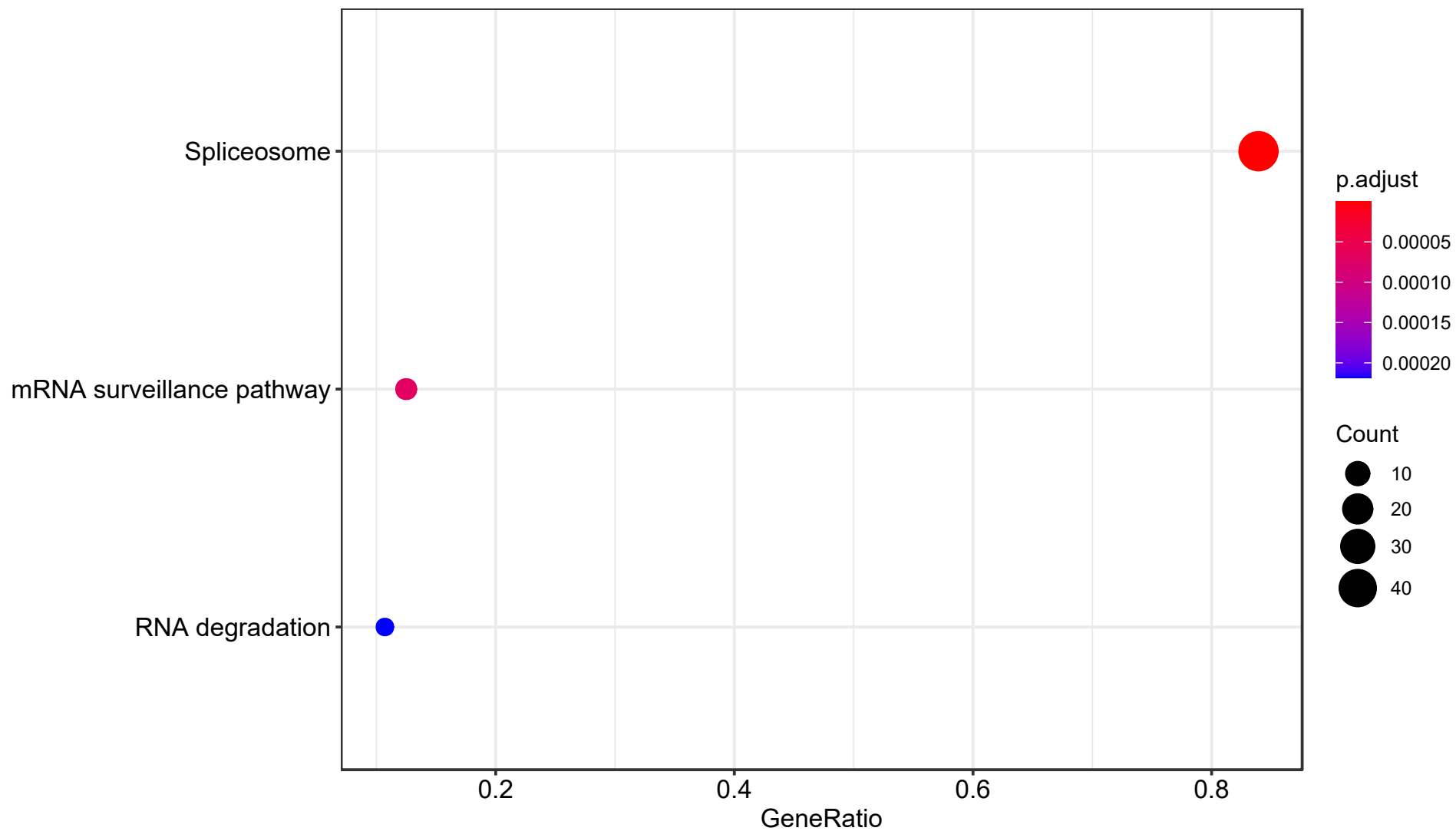

Cluster 3

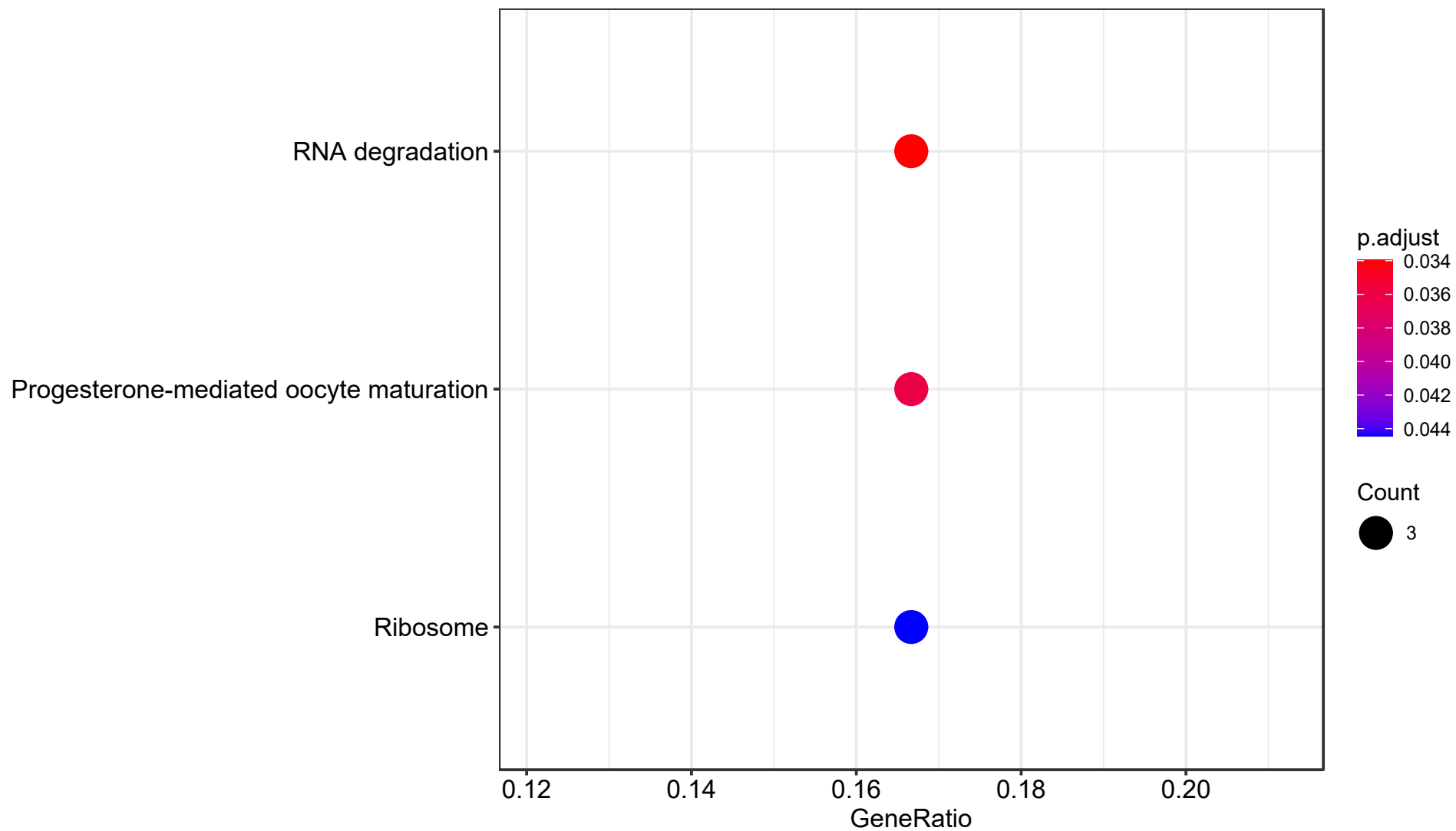

Supplement: Supplementary file 4 [file DataSheet4.PDF]

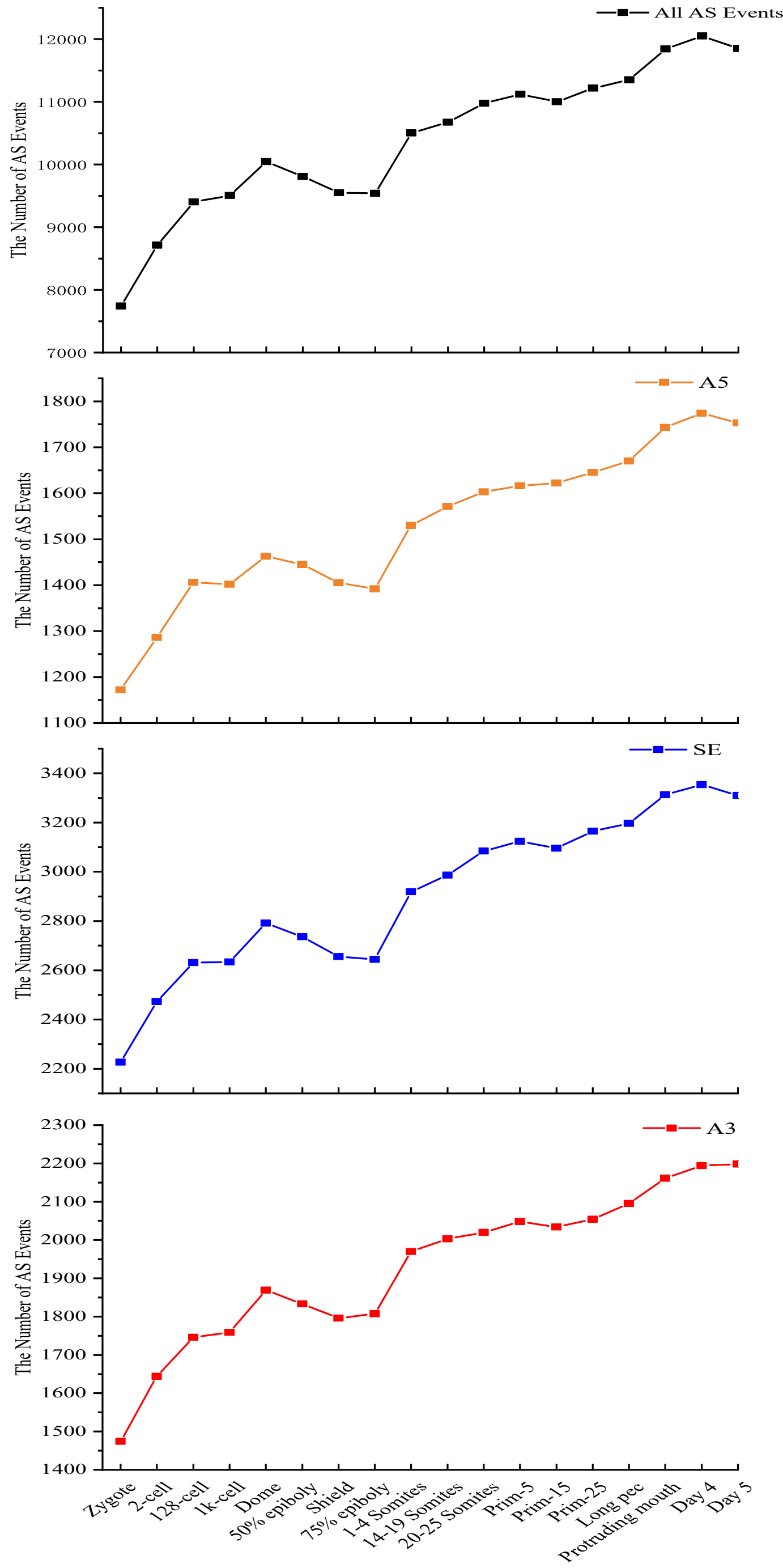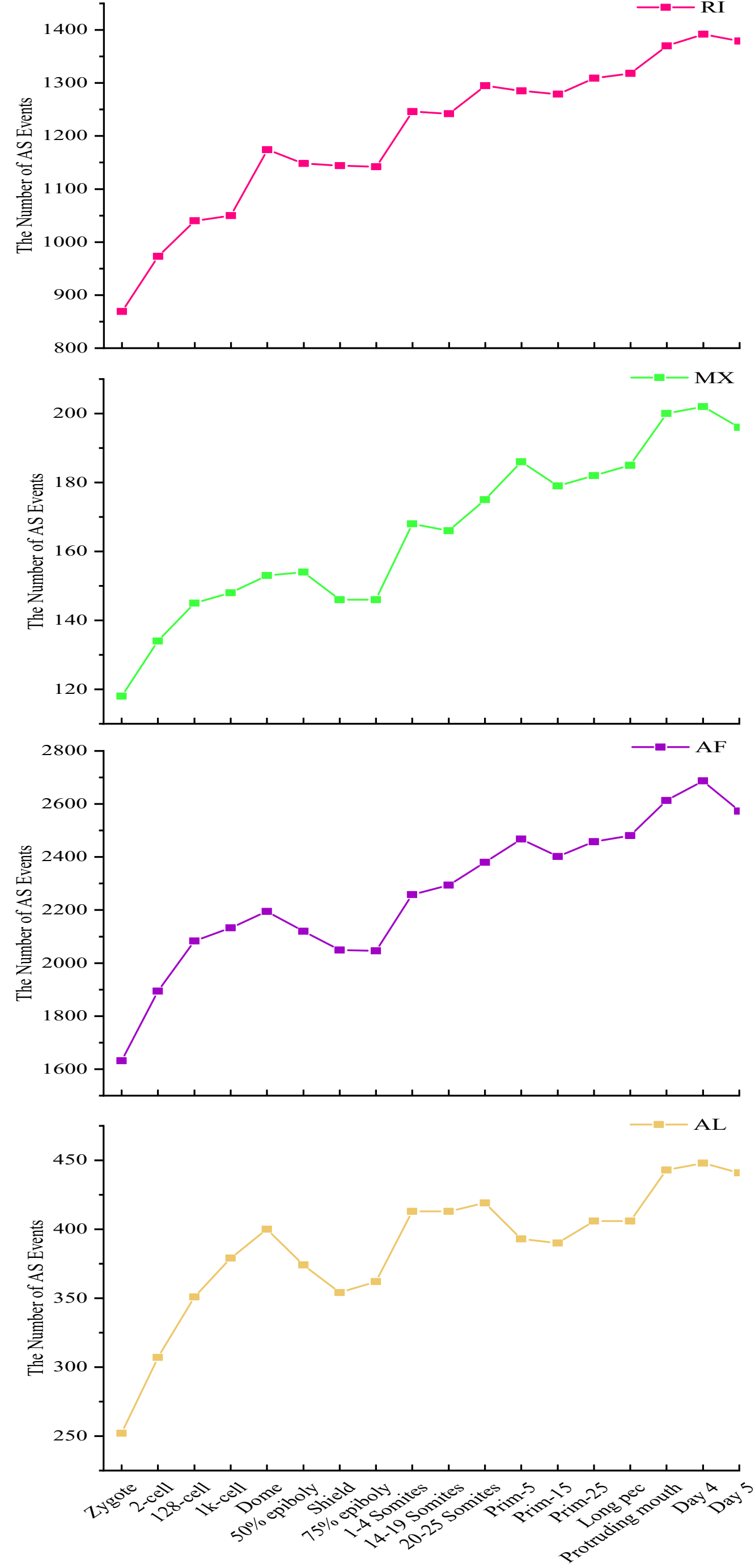

Supplement: Supplementary file 5 [file DataSheet6.PDF]

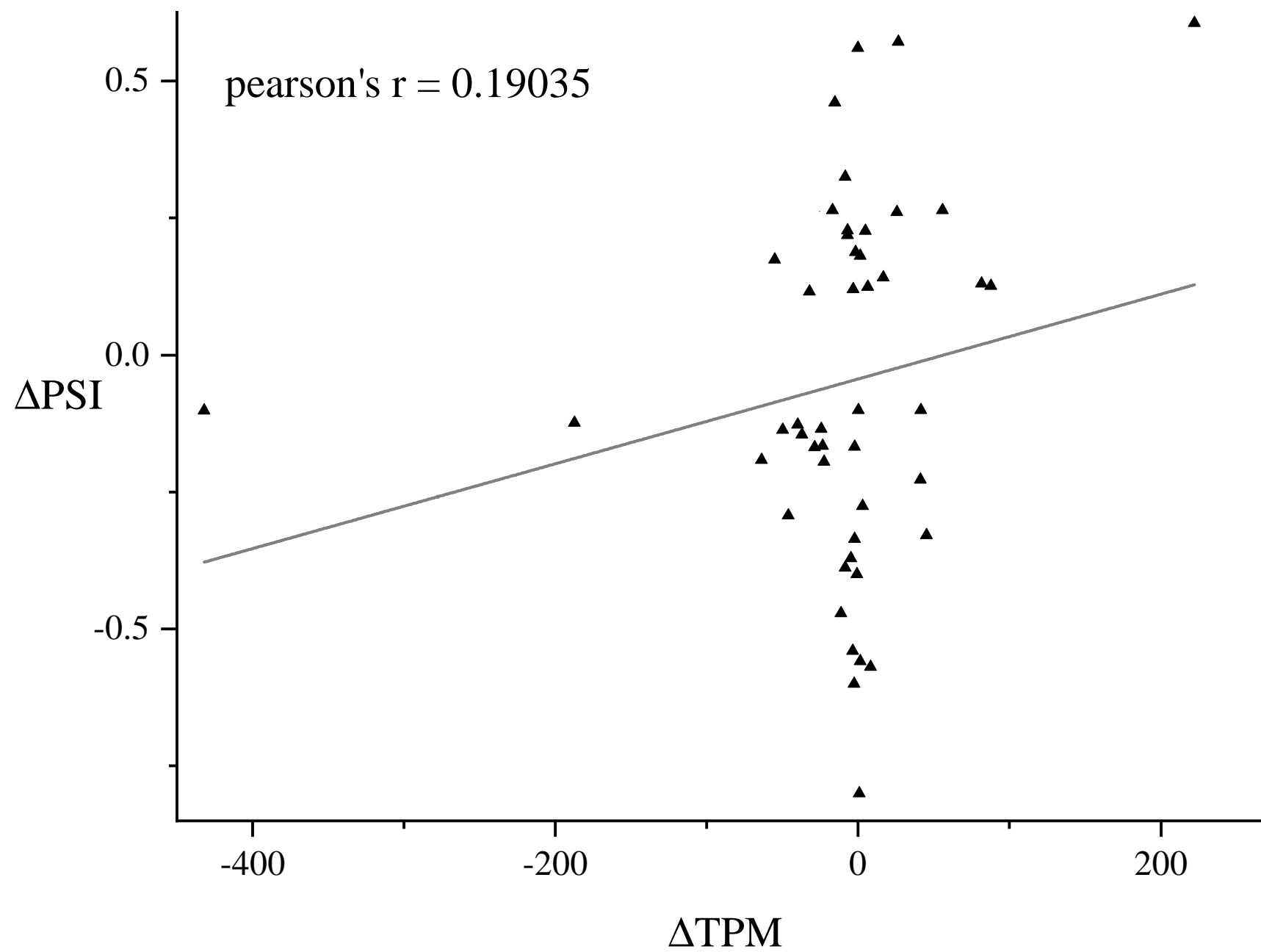

Supplement: Supplementary file 7 [file DataSheet9.PDF]

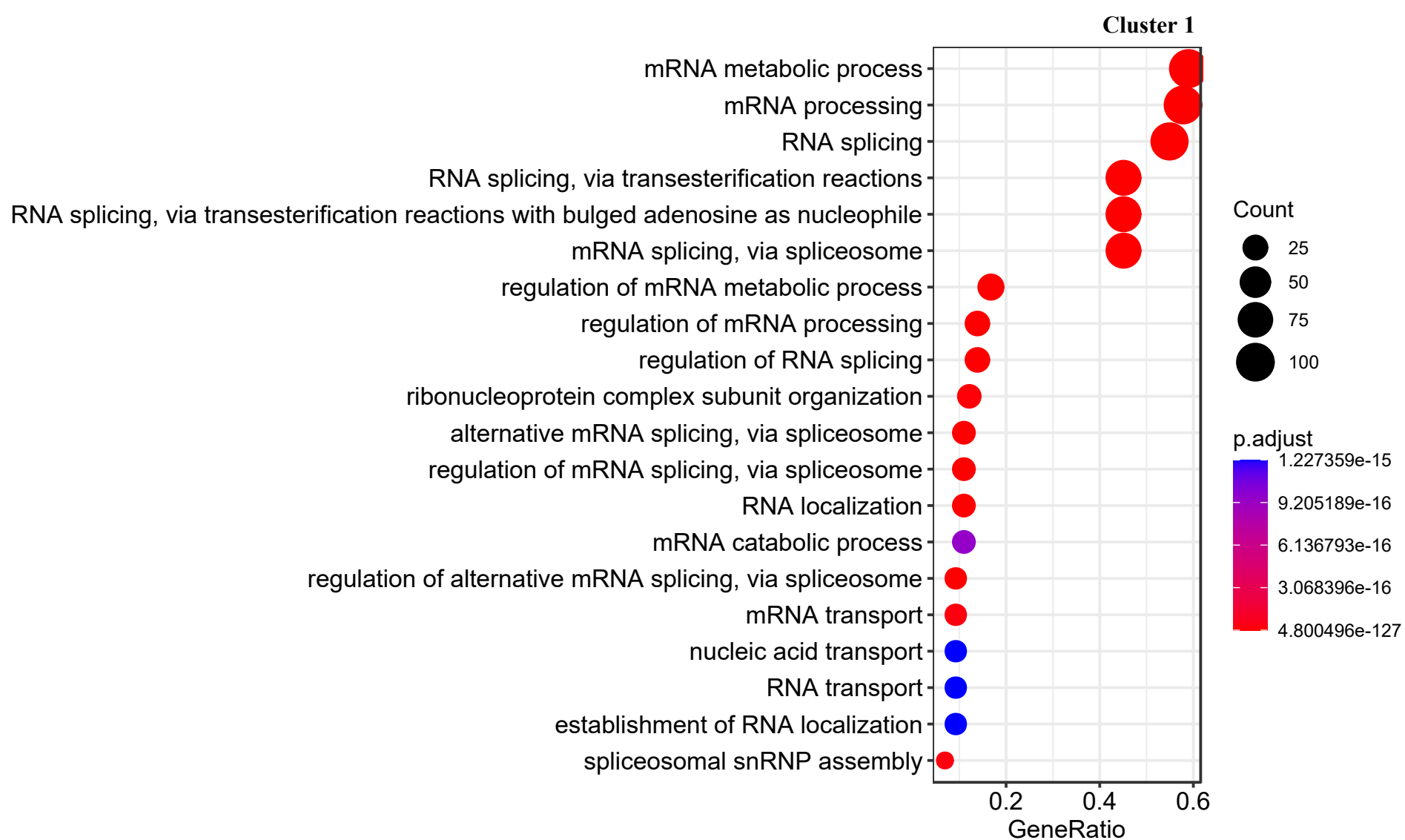

Cluster 2

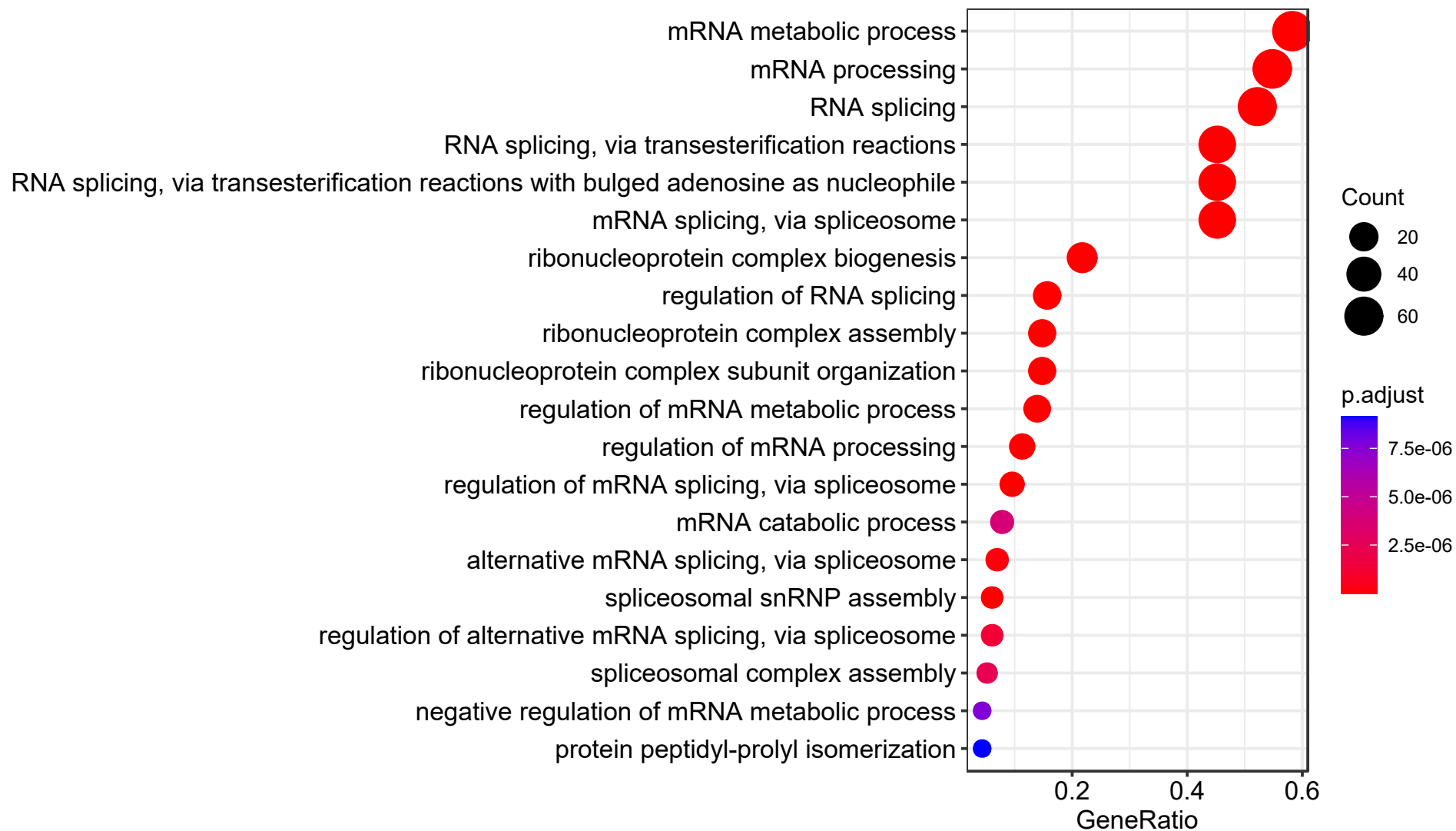

Cluster 3

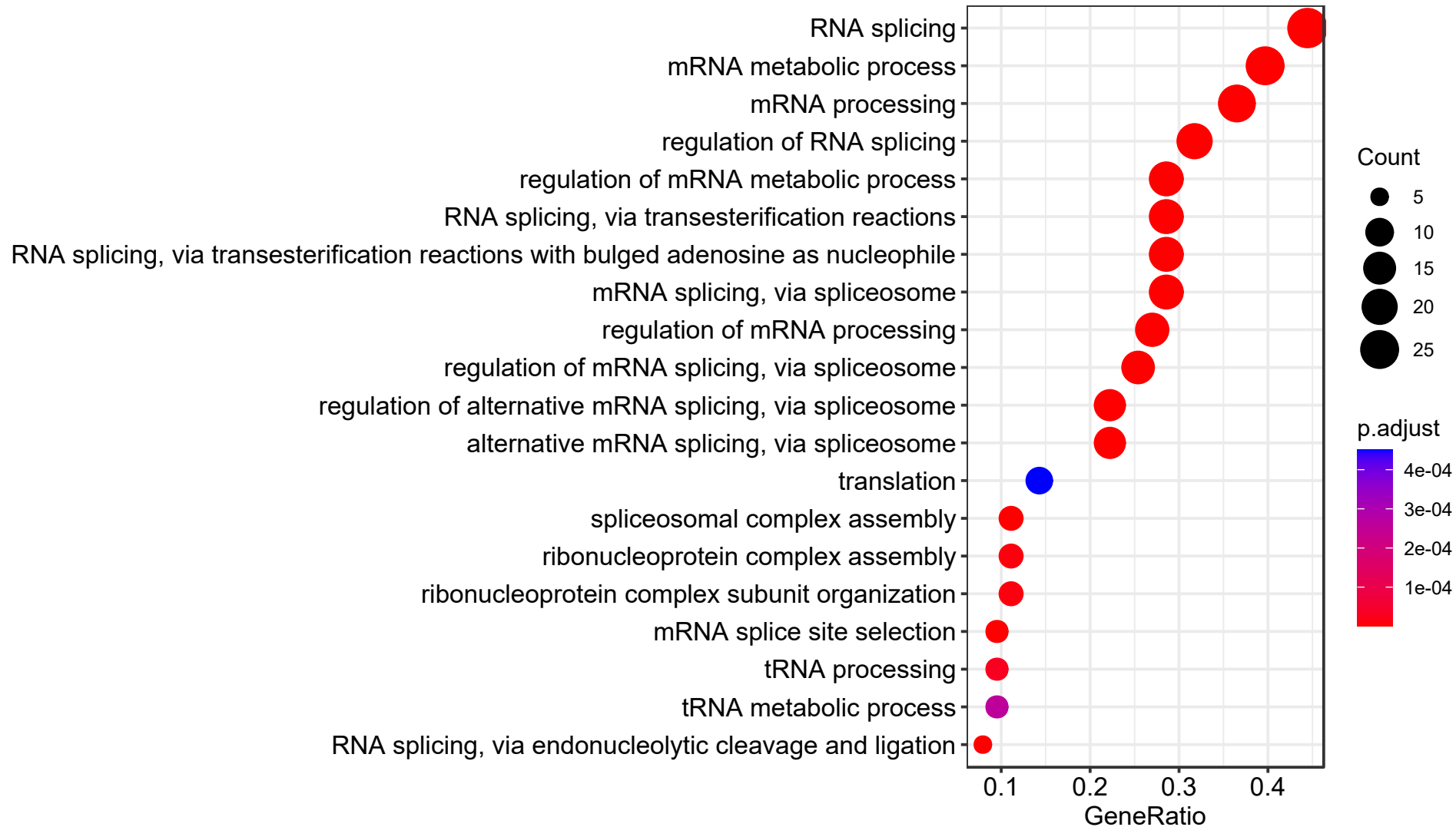

Supplement: Supplementary file 8 [file DataSheet3.PDF]

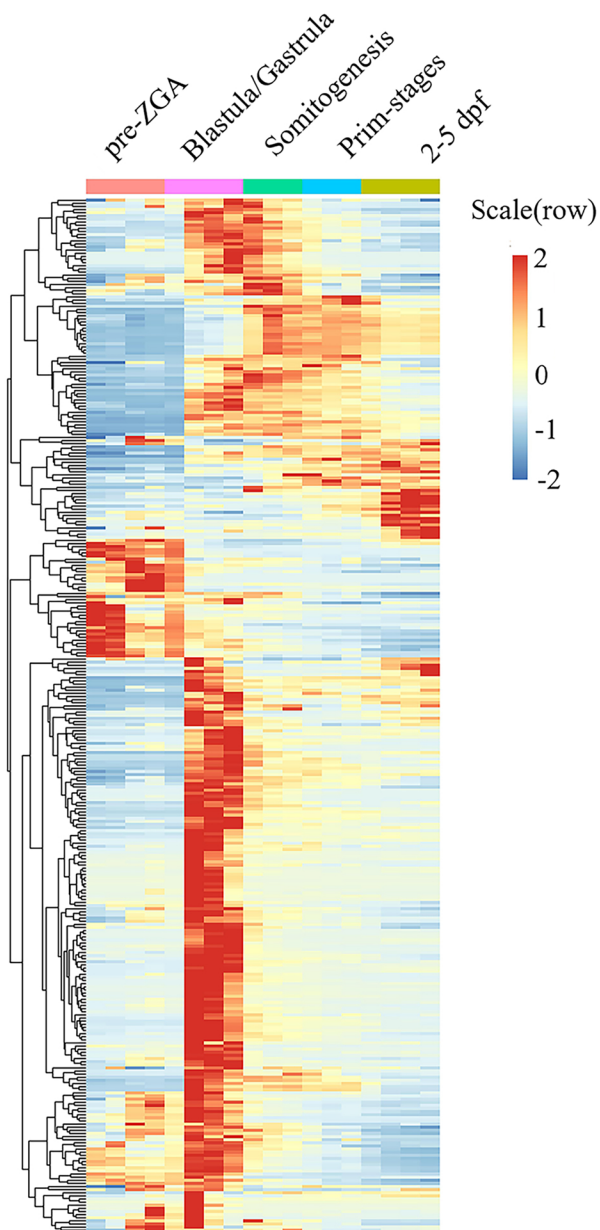

Supplement: Supplementary file 9 [file DataSheet1.PDF]

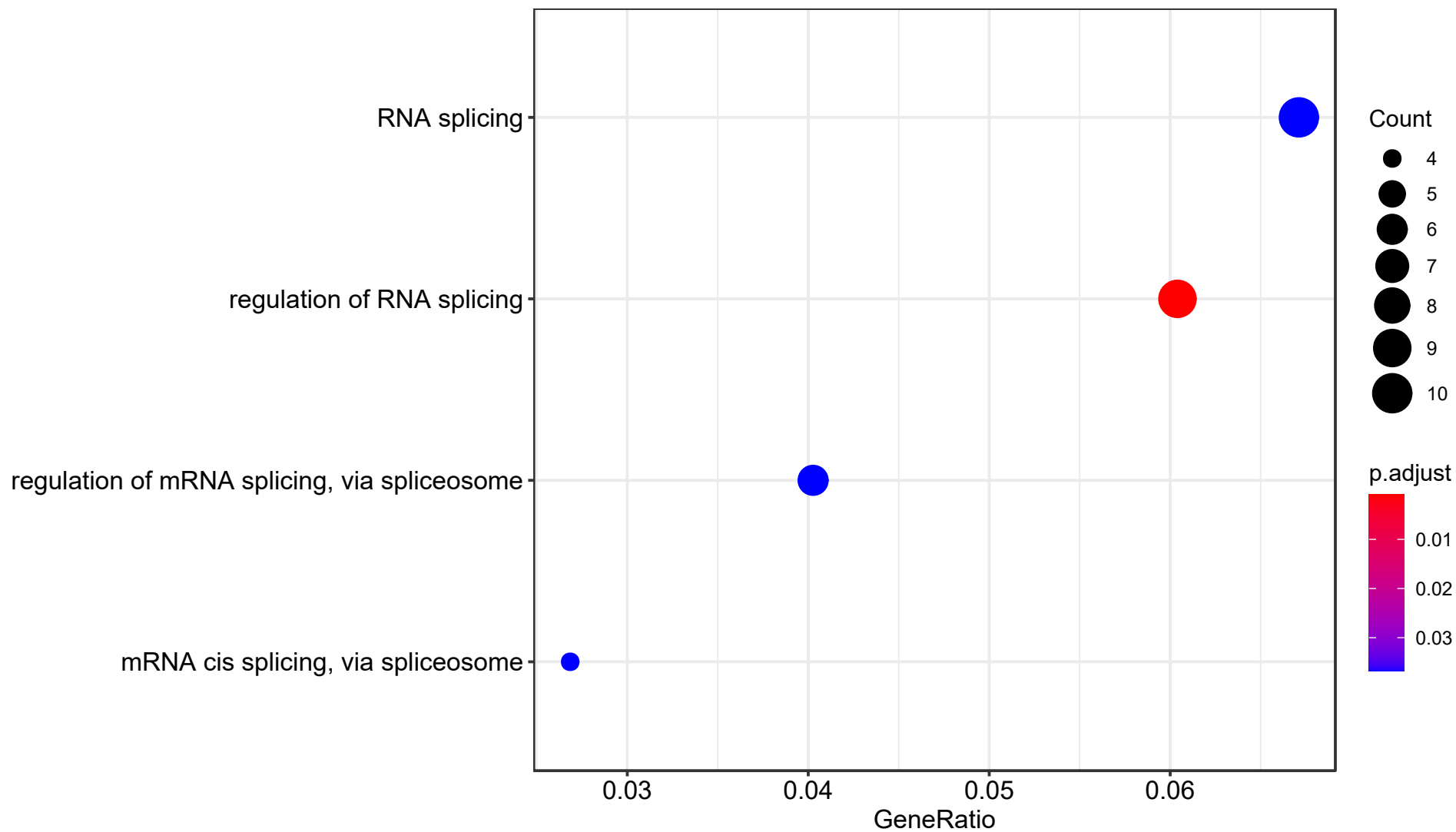

50%-epiboly\_Dome\_GO\_BP

spindle organization

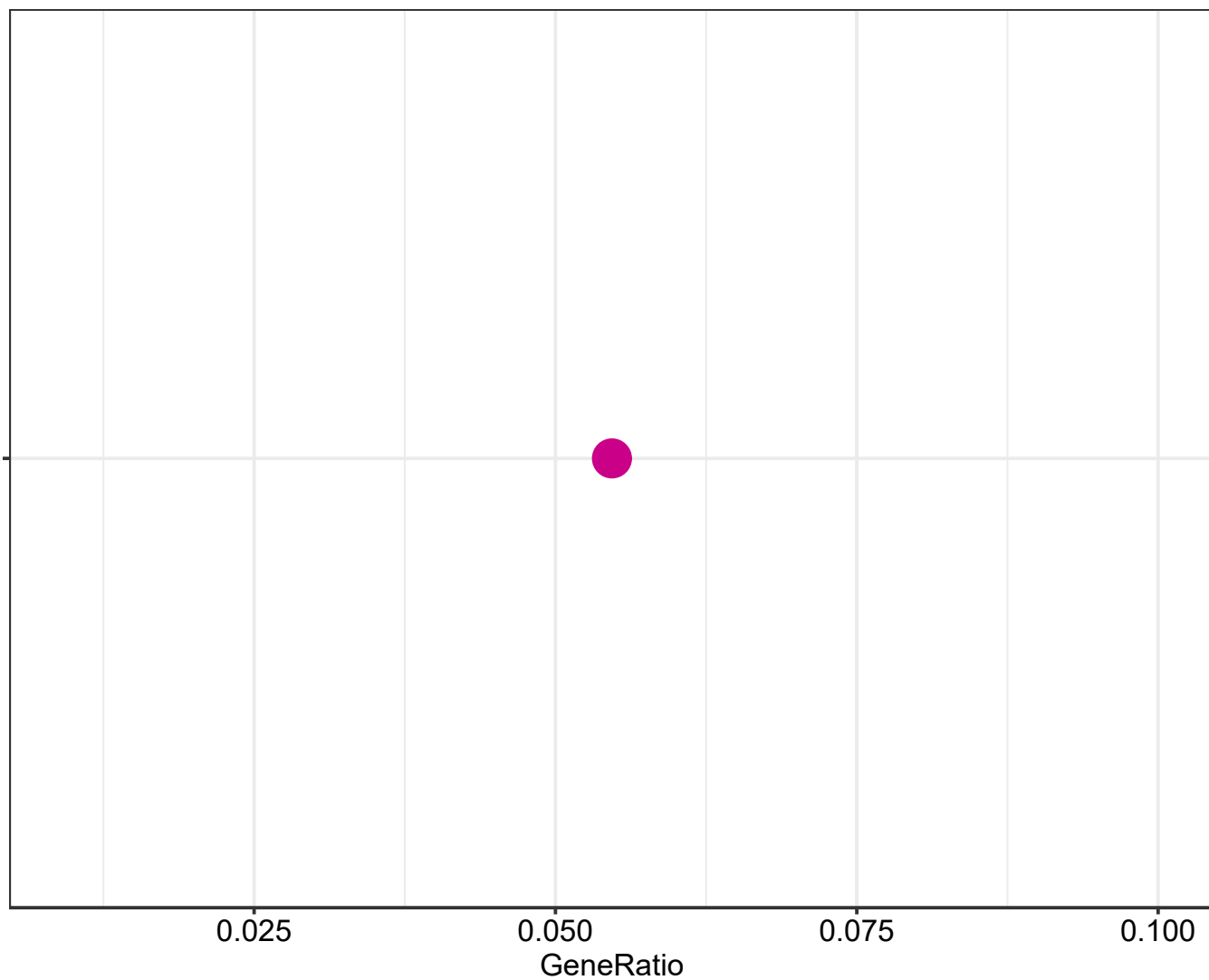

convergent extension

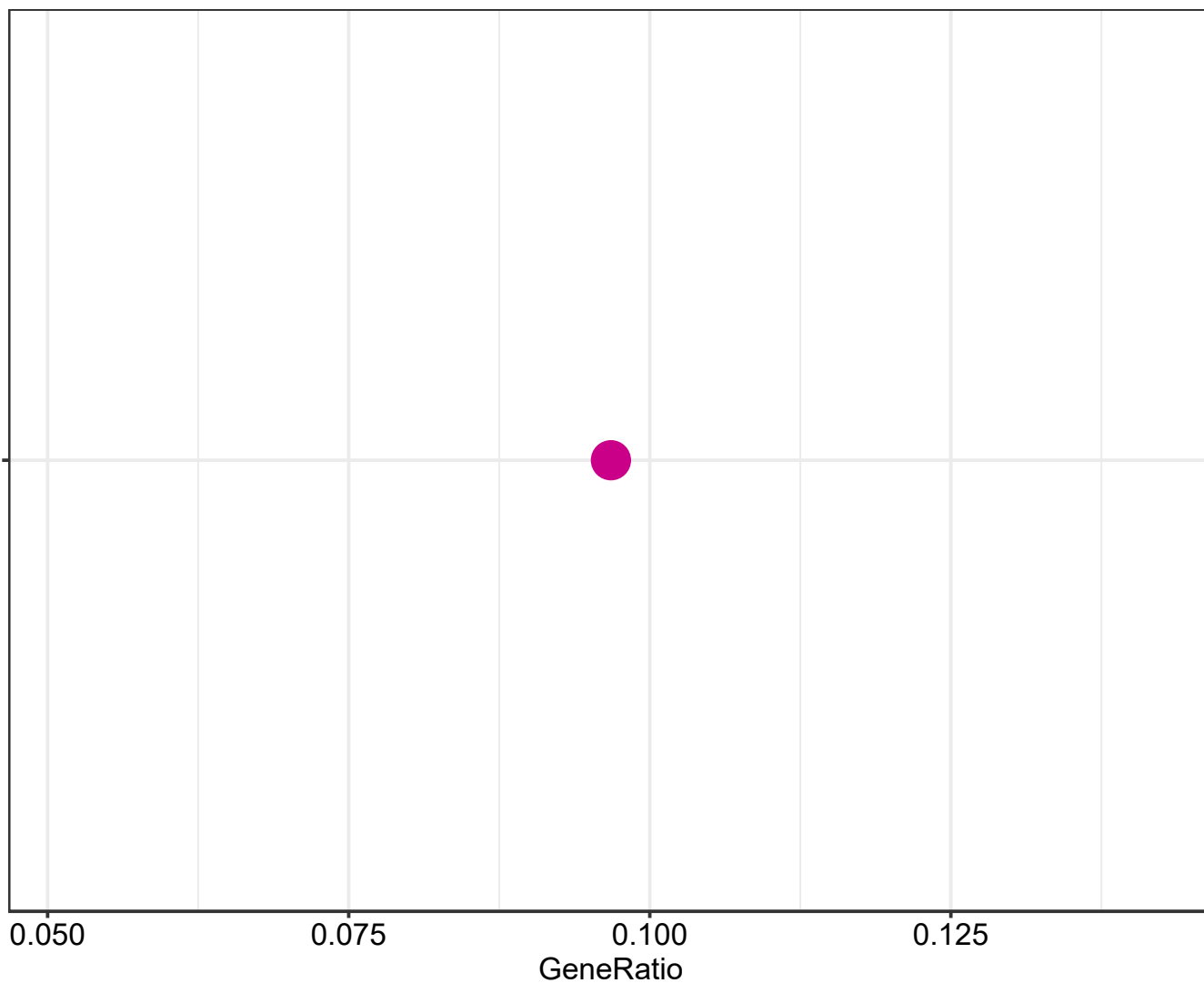

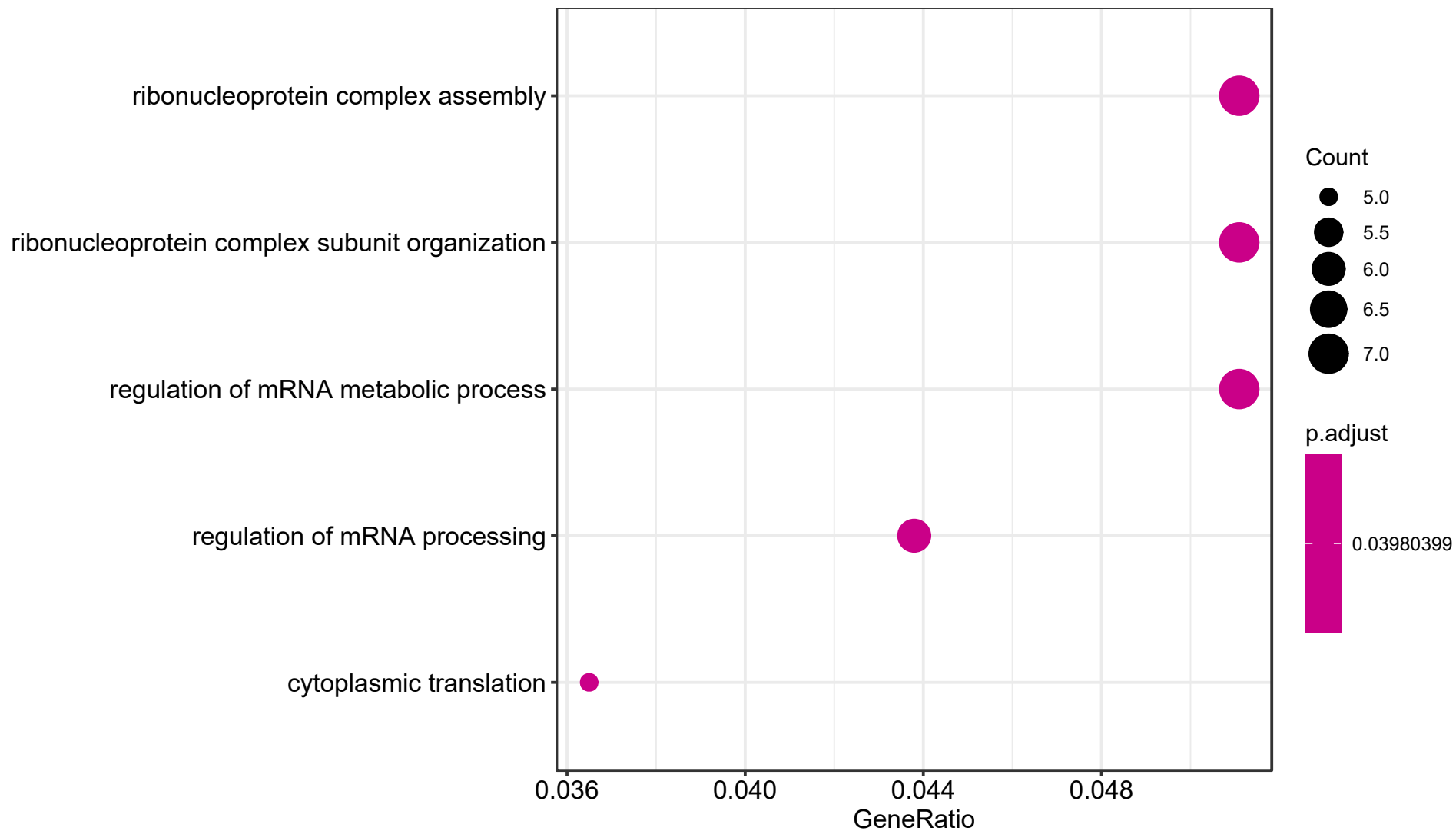

Supplement: Supplementary file 14 [file DataSheet8.PDF]
