# Supplementary material for: Temporal Dynamic Analysis of Alternative Splicing During Embryonic Development in Zebrafish
Source: Front Cell Dev Biol. 2022 Jul 8;10:879795. doi: 10.3389/fcell.2022.879795 (PMC9304896; doi:10.3389/fcell.2022.879795)
Supplement: Supplementary file 2 [file DataSheet2.pdf]

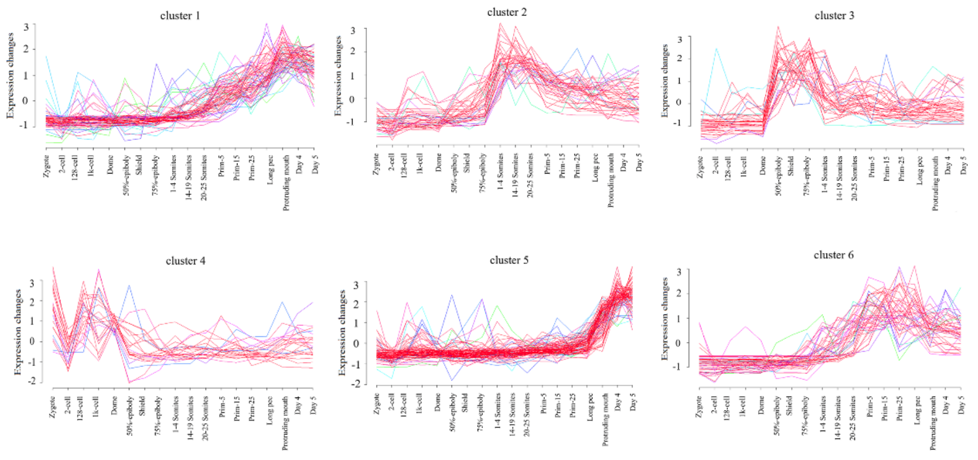

Figure S2 (A) Cluster analysis of up-regulated genes between dome and 1k-cell stages based on Mufzz.

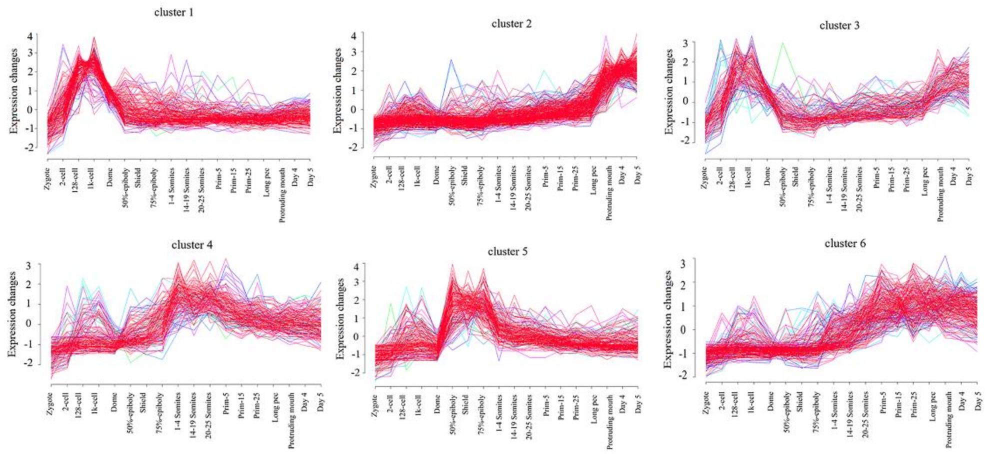

Figure S2 (B) Cluster analysis of down-regulated genes between zygote and 2-cell stages based on Mufzz.

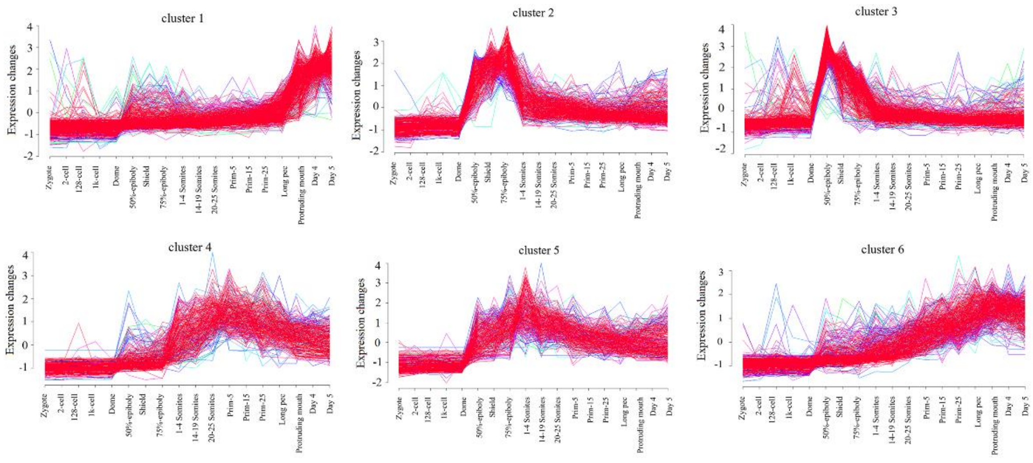

Figure S2 (C) Cluster analysis of up-regulated genes between zygote and 2-cell stages based on Mufzz.
