# Supplementary material for: Temporal Dynamic Analysis of Alternative Splicing During Embryonic Development in Zebrafish
Source: Front Cell Dev Biol. 2022 Jul 8;10:879795. doi: 10.3389/fcell.2022.879795 (PMC9304896; doi:10.3389/fcell.2022.879795)
Supplement: Supplementary file 10 [file Table5.DOCX]

**Table S5** The list of 29 splicing factors undergoing differentially alternative splicing during 8 consecutive development stages

| **Ensembl ID** | **Official ID** | **Ensembl ID** | **Official ID** | **Ensembl ID** | **Official ID** |
| --- | --- | --- | --- | --- | --- |
| ENSDARG00000007960 | hnrnpaba | ENSDARG00000008188 | sf1 | ENSDARG00000056691 | cpeb4 |
| ENSDARG00000021812 | cherp | ENSDARG00000040184 | syncrip | ENSDARG00000101744 | hug |
| ENSDARG00000040732 | elavl2 | ENSDARG00000014329 | npm1a | ENSDARG00000018814 | esrp2 |
| ENSDARG00000029248 | fubp1 | ENSDARG00000036386 | rbm4.1 | ENSDARG00000016999 | lin28a |
| ENSDARG00000059246 | hnrnpd | ENSDARG00000077860 | ankhd1 | ENSDARG00000032175 | puf60a |
| ENSDARG00000075824 | virma | ENSDARG00000102802 | brdt | ENSDARG00000005980 | sfswap |
| ENSDARG00000020482 | nono | ENSDARG00000038068 | ddx5 | ENSDARG00000074955 | nup98 |
| ENSDARG00000031907 | ptbp1b | ENSDARG00000040881 | hnrnph1 | ENSDARG00000062954 | clk2a |
| ENSDARG00000025332 | rbm28 | ENSDARG00000057691 | srsf1a | ENSDARG00000052604 | cpeb2 |
| ENSDARG00000101877 | rbm34 | ENSDARG00000039887 | c1qbp |  |  |
