# Supplementary material for: Temporal Dynamic Analysis of Alternative Splicing During Embryonic Development in Zebrafish
Source: Front Cell Dev Biol. 2022 Jul 8;10:879795. doi: 10.3389/fcell.2022.879795 (PMC9304896; doi:10.3389/fcell.2022.879795)
Supplement: Supplementary file 11 [file DataSheet5.PDF]

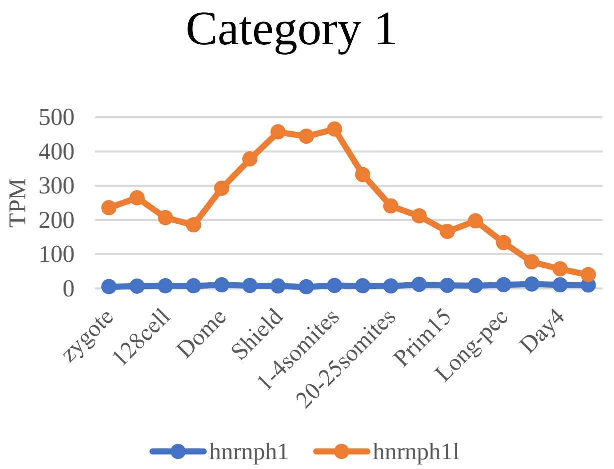
Category 1
TPM
500
400
300
200
100
0
zygote
128cell
Dome
Shield
1-4somites
20-25somites
Prim15
Long-pec
Day4
hnrnph1
hnrnph1l

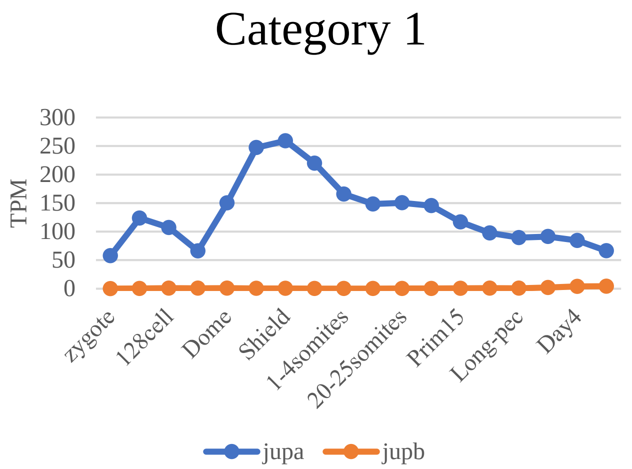
Category 1
TPM
300
250
200
150
100
50
0
zygote
128cell
Dome
Shield
1-4somites
20-25somites
Prim15
Long-pec
Day4
jupa
jupb

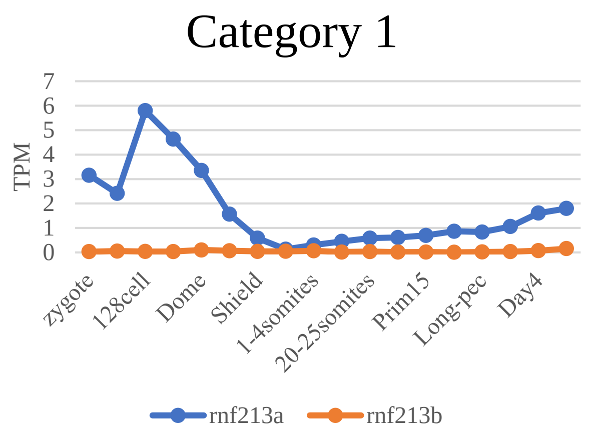
Category 1
TPM
7
6
5
4
3
2
1
0
zygote
128cell
Dome
Shield
1-4somites
20-25somites
Prim15
Long-pec
Day4
rnf213a
rnf213b

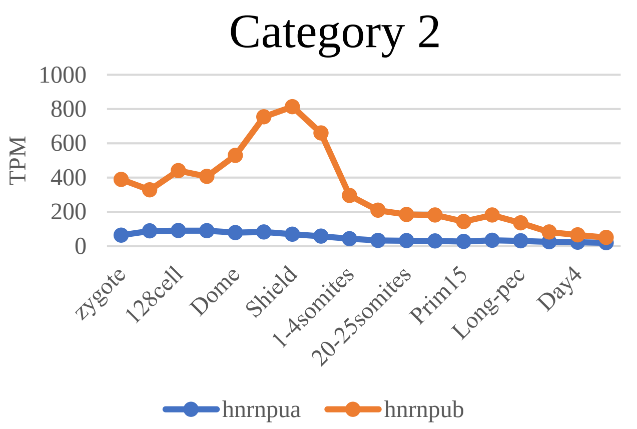
Category 2
TPM
1000
800
600
400
200
0
zygote
128cell
Dome
Shield
1-4somites
20-25somites
Prim15
Long-pec
Day4
hnrnpua
hnrnpub

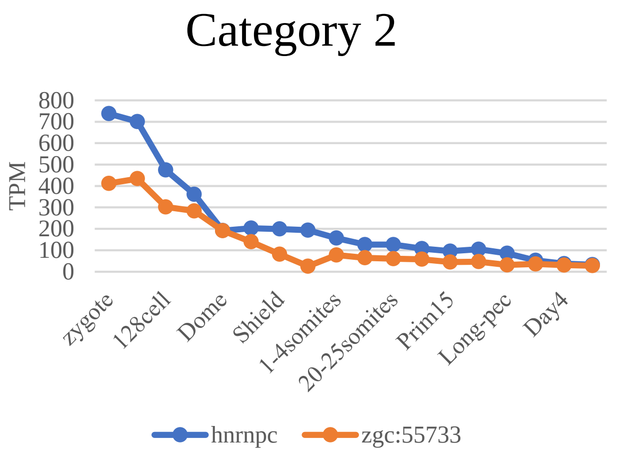
Category 2
TPM
800
700
600
500
400
300
200
100
0
zygote
128cell
Dome
Shield
1-4somites
20-25somites
Prim15
Long-pec
Day4
hnrnpc
zgc:55733

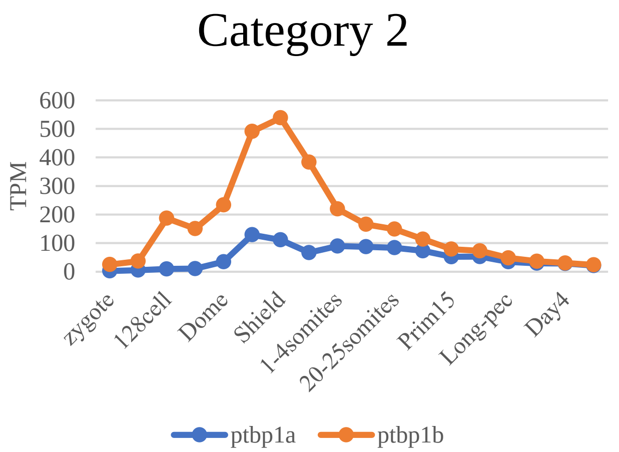
Category 2
TPM
600
500
400
300
200
100
0
zygote
128cell
Dome
Shield
1-4somites
20-25somites
Prim15
Long-pec
Day4
ptbp1a
ptbp1b

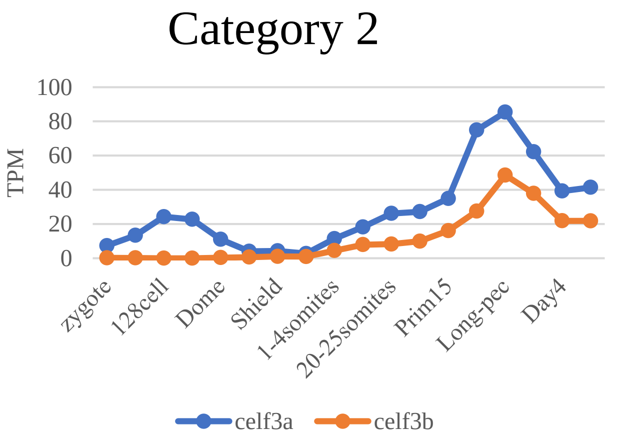
Category 2
TPM
100
80
60
40
20
0
zygote
128cell
Dome
Shield
1-4somites
20-25somites
Prim15
Long-pec
Day4
celf3a
celf3b

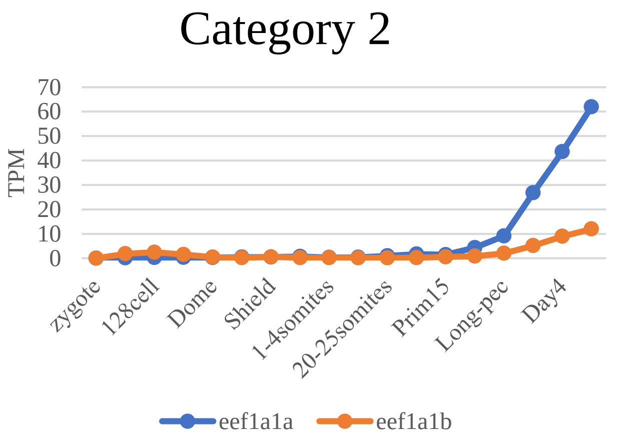
Category 2
TPM
70
60
50
40
30
20
10
0
zygote
128cell
Dome
Shield
1-4somites
20-25somites
Prim15
Long-pec
Day4
eef1a1a
eef1a1b

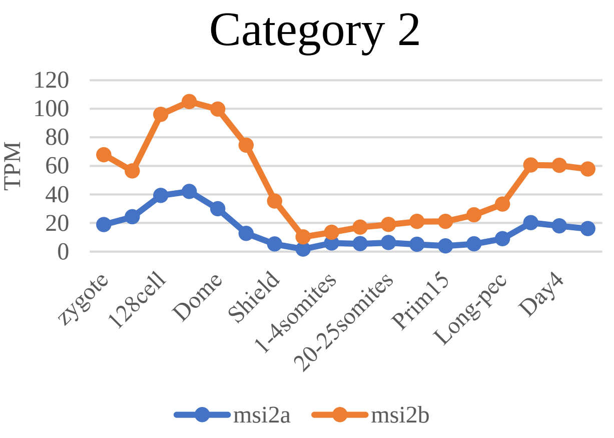
Category 2
TPM
120
100
80
60
40
20
0
zygote
128cell
Dome
Shield
1-4somites
20-25somites
Prim15
Long-pec
Day4
msi2a
msi2b

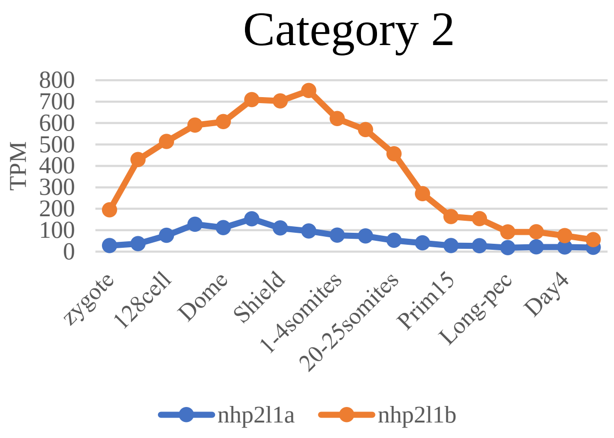
Category 2
TPM
800
700
600
500
400
300
200
100
0
zygote
128cell
Dome
Shield
1-4somites
20-25somites
Prim15
Long-pec
Day4
nhp2l1a
nhp2l1b

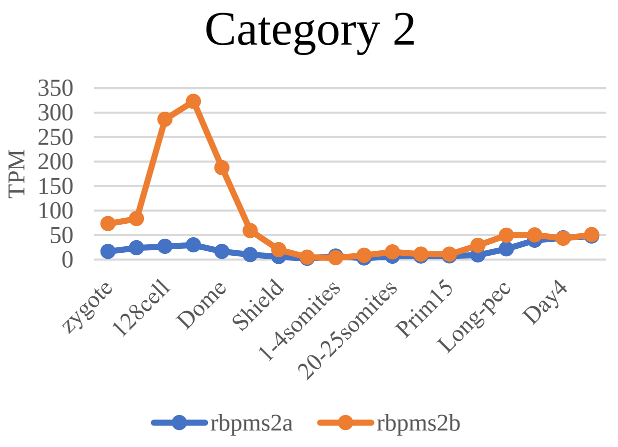
Category 2
TPM
350
300
250
200
150
100
50
0
zygote
128cell
Dome
Shield
1-4somites
20-25somites
Prim15
Long-pec
Day4
rbpms2a
rbpms2b

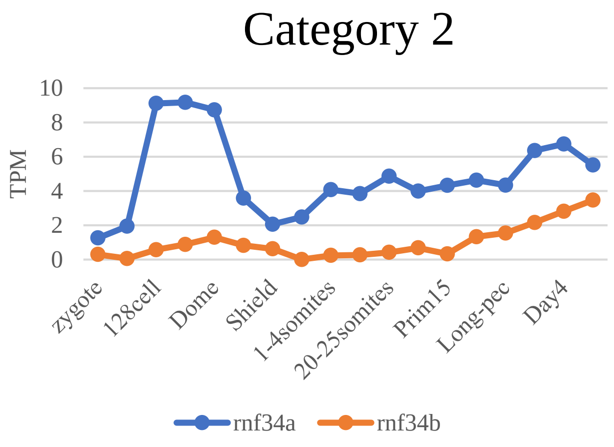
Category 2
TPM
10
8
6
4
2
0
zygote
128cell
Dome
Shield
1-4somites
20-25somites
Prim15
Long-pec
Day4
rnf34a
rnf34b

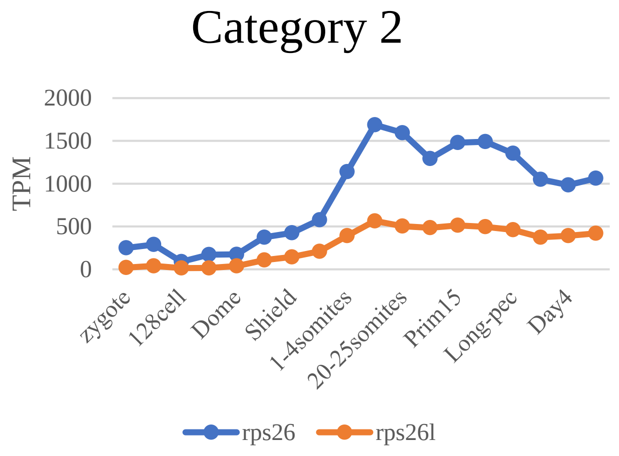
Category 2
TPM
2000
1500
1000
500
0
zygote
128cell
Dome
Shield
1-4somites
20-25somites
Prim15
Long-pec
Day4
rps26
rps26l

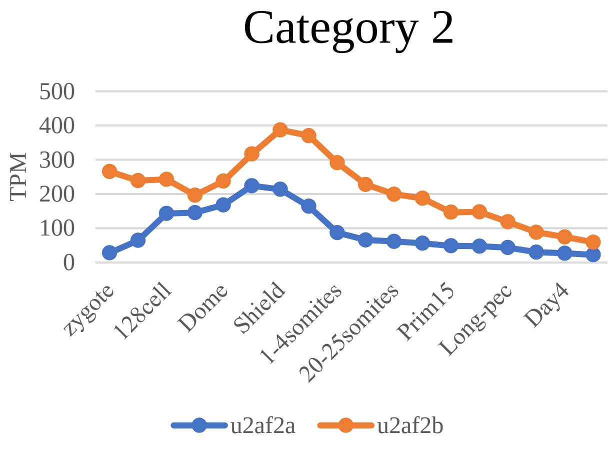
Category 2
TPM
500
400
300
200
100
0
zygote
128cell
Dome
Shield
1-4somites
20-25somites
Prim15
Long-pec
Day4
u2af2a
u2af2b

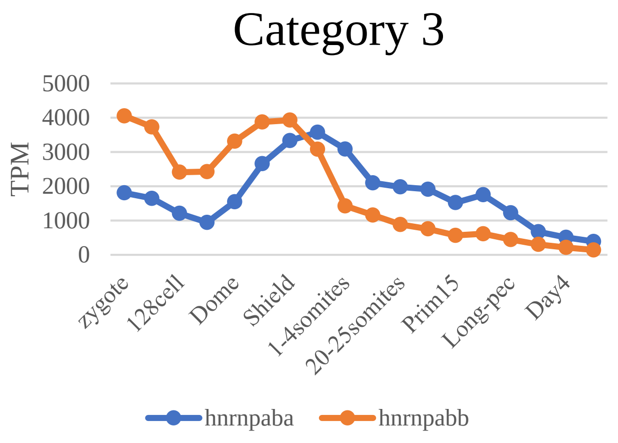
Category 3
TPM
5000
4000
3000
2000
1000
0
zygote
128cell
Dome
Shield
1-4somites
20-25somites
Prim15
Long-pec
Day4
hnrnpaba
hnrnpabb

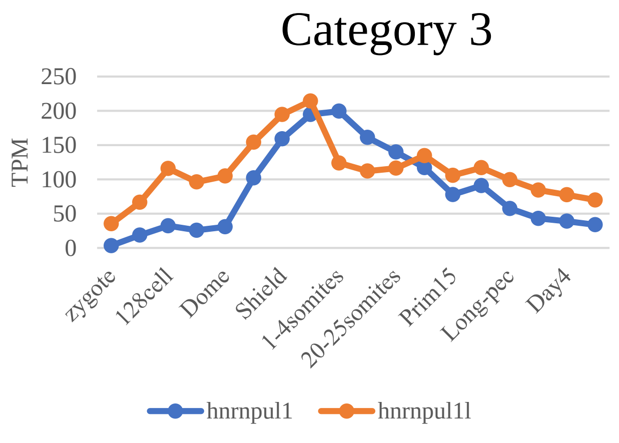
Category 3
TPM
250
200
150
100
50
0
zygote
128cell
Dome
Shield
1-4somites
20-25somites
Prim15
Long-pec
Day4
hnrnpul1
hnrnpul1l

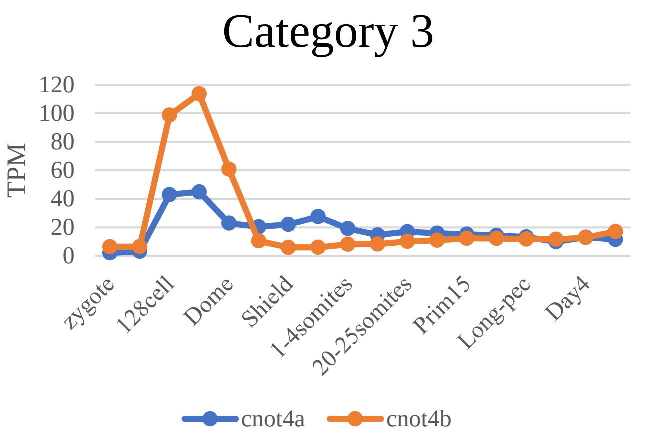
Category 3
TPM
120
100
80
60
40
20
0
zygote
128cell
Dome
Shield
1-4somites
20-25somites
Prim15
Long-pec
Day4
cnot4a
cnot4b

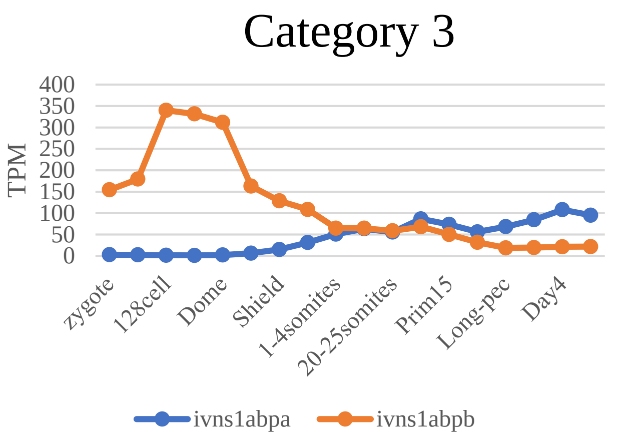
Category 3
TPM
400
350
300
250
200
150
100
50
0
zygote
128cell
Dome
Shield
1-4somites
20-25somites
Prim15
Long-pec
Day4
ivns1abpa
ivns1abpb

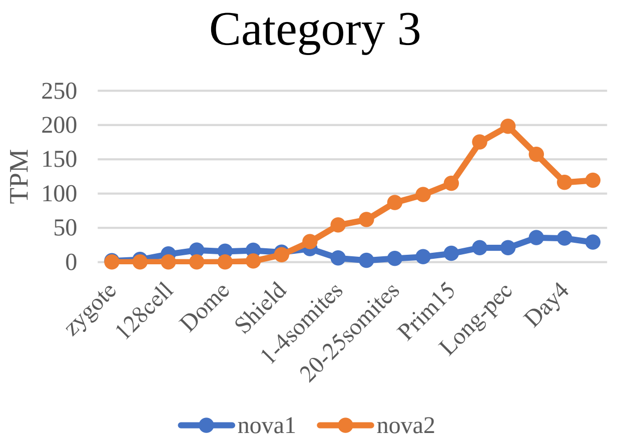
Category 3
TPM
250
200
150
100
50
0
zygote
128cell
Dome
Shield
1-4somites
20-25somites
Prim15
Long-pec
Day4
nova1
nova2

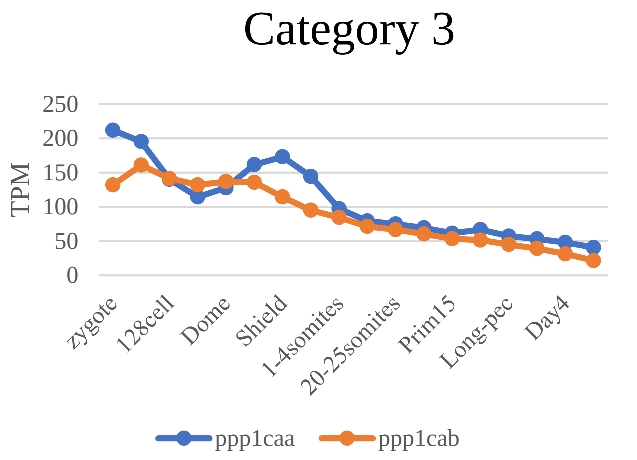
Category 3
TPM
250
200
150
100
50
0
zygote
128cell
Dome
Shield
1-4somites
20-25somites
Prim15
Long-pec
Day4
ppp1caa
ppp1cab

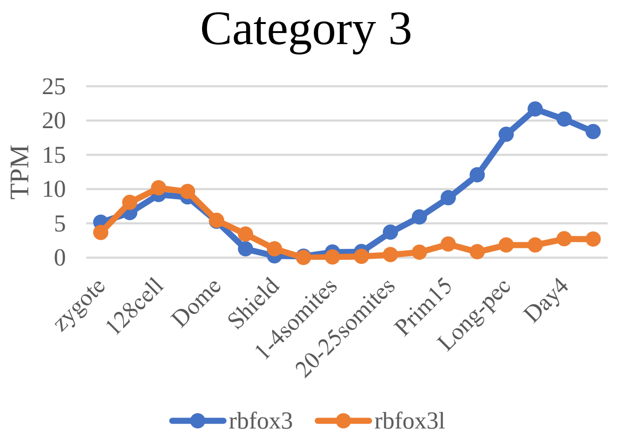
Category 3
TPM
25
20
15
10
5
0
zygote
128cell
Dome
Shield
1-4somites
20-25somites
Prim15
Long-pec
Day4
rbfox3
rbfox3l

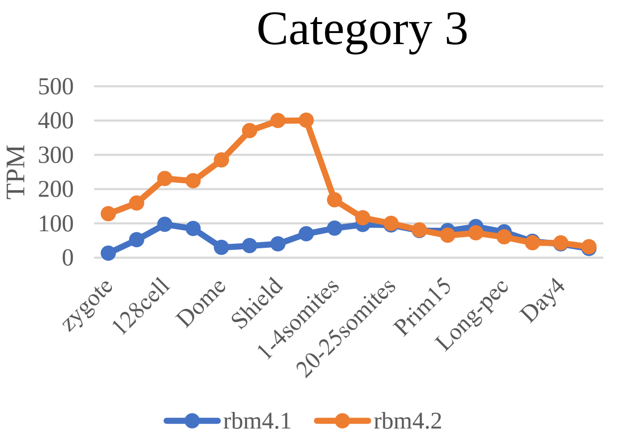
Category 3
TPM
500
400
300
200
100
0
zygote
128cell
Dome
Shield
1-4somites
20-25somites
Prim15
Long-pec
Day4
rbm4.1
rbm4.2

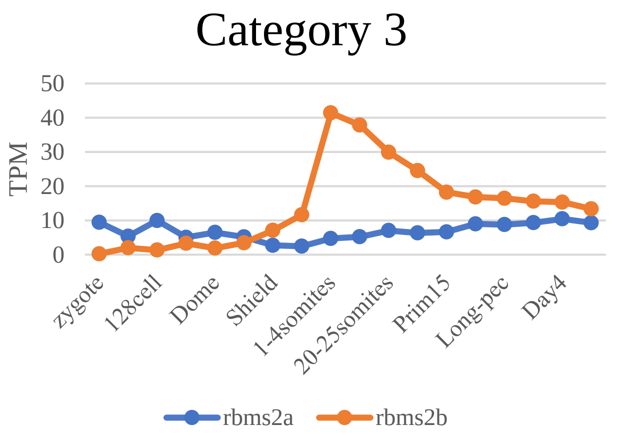
Category 3
TPM
50
40
30
20
10
0
zygote
128cell
Dome
Shield
1-4somites
20-25somites
Prim15
Long-pec
Day4
rbms2a
rbms2b

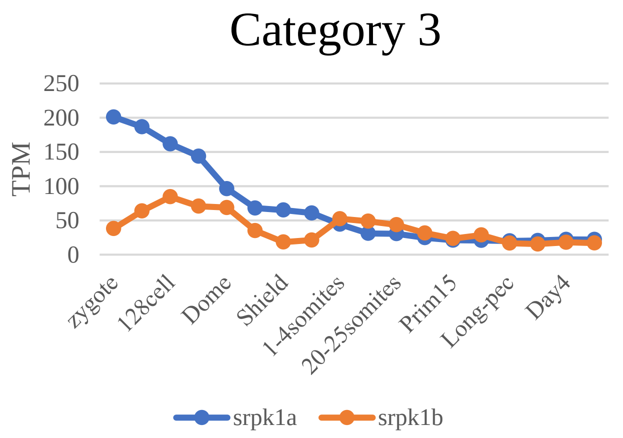
Category 3
TPM
250
200
150
100
50
0
zygote
128cell
Dome
Shield
1-4somites
20-25somites
Prim15
Long-pec
Day4
srpk1a
srpk1b
